# Exogenous methionine contributes to reversing the resistance of *Streptococcus suis* to macrolides

**DOI:** 10.1128/spectrum.02803-23

**Published:** 2024-01-17

**Authors:** Chun-Liu Dong, Tong Wu, Yue Dong, Qian-Wei Qu, Xue-Ying Chen, Yan-Hua Li

**Affiliations:** 1 College of Veterinary Medicine, Northeast Agricultural University, Harbin, Heilongjiang, China; 2 Heilongjiang Key Laboratory for Animal Disease Control and Pharmaceutical Development, Harbin, Heilongjiang, China; Michigan State University, East Lansing, Michigan, USA

**Keywords:** *Streptococcus suis*, methionine, methyl metabolism, macrolides, metal starvation

## Abstract

*Streptococcus suis* (*S. suis*) has been increasingly recognized as a porcine zoonotic pathogen that threatens the health of both pigs and humans. Multidrug-resistant *Streptococcus suis* is becoming increasingly prevalent, and novel strategies to treat bacterial infections caused by these organisms are desperately needed. In the present study, an untargeted metabolomics analysis showed that the significant decrease in methionine content and the methionine biosynthetic pathway were significantly affected by the Kyoto Encyclopedia of Genes and Genomes pathway enrichment analysis in drug-resistant *S. suis*. The addition of L-methionine restored the bactericidal activity of macrolides, doxycycline, and ciprofloxacin on *S. suis in vivo* and *in vitro*. Further studies showed that the exogenous addition of methionine affects methionine metabolism by reducing S-adenosylmethionine synthetase activity and the contents of S-adenosylmethionine, S-adenosyl homocysteine, and S-ribose homocysteine. Methionine can decrease the total methylation level and methylesterase activity in multidrug resistant *S. suis*. The drug transport proteins and efflux pump genes were significantly downregulated in *S. suis* by exogenous L-methionine. Moreover, the exogenous addition of methionine can reduce the survival of *S. suis* by affecting oxidative stress and metal starvation in bacteria. Thus, L-methionine may influence the development of resistance in *S. suis* through methyl metabolism and metal starvation. This study provides a new perspective on the mitigation of drug resistance in *S. suis*.

**IMPORTANCE:**

Bacterial antibiotic resistance has become a severe threat to human and animal health. Increasing the efficacy of existing antibiotics is a promising strategy against antibiotic resistance. Here, we report that L-methionine enhances the efficacy of macrolides, doxycycline, and ciprofloxacin antibiotics in killing *Streptococcus suis*, including multidrug-resistant pathogens. We investigated the mechanism of action of exogenous methionine supplementation in restoring macrolides in *Streptococcus suis* and the role of the methionine cycle pathway on methylation levels, efflux pump genes, oxidative stress, and metal starvation in *Streptococcus suis*. It provides a theoretical basis for the rational use of macrolides in clinical practice and also identifies a possible target for restoring drug resistance in *Streptococcus suis*.

## INTRODUCTION

*Streptococcus suis* (*S. suis*) is an important bacterial pathogen that can cause meningitis, septicemia, and even sudden death in humans and pigs (1), which has resulted in huge economic losses to the pig farming industry and threatened the public health and safety of the community (2). One of the main causes of persistent *S. suis* infections is the varying degrees of drug resistance of clinically isolated strains to drugs, including macrolides, fluoroquinolones, and tetracyclines (3–5). Under the pressure of long-term selection of antimicrobial drugs, bacteria become resistant through adaptive resistance, genetic mutation, heterogeneous resistance, and many other ways, resulting in the clinical failure of antimicrobial drug therapy (6). However, the spread of drug-resistant bacteria and the failure to treat some bacterial diseases cannot be fully explained. As the mechanisms of bacterial drug resistance have been investigated, the metabolism of bacteria and the products in their metabolic pathways have been found to be directly related to their drug resistance (7). When bacteria are exposed to drugs, their protein networks are altered, resulting in the corresponding metabolic pathways being altered, which makes the bacteria less susceptible to antimicrobial drugs and promotes the production of drug-resistant and persistent bacteria (8). Moreover, bacterial metabolism and the transmission of signaling molecules are also related to their drug resistance (9).

Amino acids are used as an essential part of protein composition and are the physiological core of all living cells. Amino acids are substrates involved in protein biosynthesis and are also used as an important source of carbon and nitrogen in physiological metabolism (10). Moreover, amino acids are also involved in bacterial cell wall biosynthesis and maintenance of bacterial homeostasis (11). Recent studies have shown that bacteria can maintain their own survival and resist various exogenous stimuli by regulating the dynamic balance of amino acids (12). Among them, sugar metabolism, amino acid metabolism, and components of metabolic pathways being altered can affect the susceptibility of bacteria to drugs (13). Metabolites such as glycine were added exogenously to significantly increase the susceptibility of various bacteria (*Escherichia coli*, *Klebsiella pneumoniae*, *Staphylococcus aureus,* and *Pseudomonas aeruginosa*) to ampicillin and kanamycin by regulating purine metabolism, TCA cycle, and glycine, serine and threonine metabolic pathways (14).

The methionine (MET) biosynthetic pathway, carried by almost all prokaryotes, has been studied as an adjuvant for a variety of broad-spectrum antibiotics (15). MET is essential for the biosynthesis of bacterial proteins and is required for the initiation and extension phases of translation (16). Meanwhile, MET is a precursor of the important methyl donor S-adenosylmethionine (SAM), which is involved in the activated methyl cycle and is required for DNA methylation, protein methylation, and polyamine biosynthesis (17). In the methyl cycle, SAM is the main methyl donor and forms S-adenosyl homocysteine (SAH) catalyzed by S-adenosylmethionine synthetase (SAMs). Then, SAH is converted into homocysteine (HCY) through a two-step process (18). It has been shown that after the gene expressing DNA cytosine methyltransferase was knocked out in *E. coli*, the drug tolerance transporter protein showed an overexpressed state and was tolerant to ethidium bromide (19). However, back-complementation of the gene restored the susceptibility of *E. coli* to ethidium bromide (19), suggesting that altering the methyl cycle affects methylation and has a modulating effect on bacterial drug resistance. However, there are few studies on whether *S. suis* resistance is associated with changes in physiological metabolism. A recent study by Li et al. investigated how *S. suis* serine/threonine kinases can affect the virulence and drug resistance of *S. suis* (20). We found that L-serine can reduce the survival rate of resistant strains of *S. suis* by increasing the production of reactive oxygen species (ROS) (21). In addition, gallic acid can restore the sulfonamide sensitivity of multidrug-resistant *S. suis* by regulating the expression of dihydrofolate reductase (22).

At the same time, the oxidative stress and metal starvation encountered by *S. suis* during the infection process cannot be ignored. The regulation of oxidative stress and metal starvation responses by ferric uptake regulator (Fur)-like proteins is also important for the survival of *S. suis in vivo* (23). We investigated whether exogenous metabolites have the potential to restore the susceptibility of *S. suis* to antimicrobial drugs by altering its physiological metabolic profile. Here, we describe in detail the metabolic changes in *S. suis* following antibiotic resistance and demonstrate that L-methionine (L-met) affects the methyl cycle of *S. suis* and, in the presence of metabolites, inhibits methyl metabolism and affects *perR*-regulated oxidative stress, restoring bacterial susceptibility to antibacterial drugs.

## RESULTS

### Analysis of *Streptococcus suis* resequencing data

We conducted an analysis of the resequencing data for *S. suis* strains T-I-8, T-I-64, and T-I-256. In the case of *S. suis* T-I-8, we identified only one single nucleotide polymorphism (SNP). However, this SNP was located downstream/upstream and appeared to have little functional significance. In the strain *S. suis* T-I-64, seven SNPs were identified, all of which are nonsynonymous and are located in the coding sequence (CDS) region. We annotated seven candidate genes that play important roles in various biological processes, including ribosomal protein synthesis, fatty acid biosynthesis, magnesium ion transport, ATP binding, antibiotic resistance, and oxidative stress (Table 1). The results showed that nine high-quality SNPs based on whole-genome resequencing were discovered in *S. suis* T-I-256 (seven nonsynonymous, one stop-gain, and one noncoding), among which eight SNPs were located in the CDS region. Out of the nine SNPs, only three were distinct from those observed in *S. suis* T-I-64. Two of these SNPs were associated with aquaporin family proteins and ABC transporter ATP-binding proteins, while one had no apparent function. Furthermore, we identified 50 indels (insertions or deletions) in the genome of *S. suis* T-I-8 when compared to the reference genome (NC_012925.1). Remarkably, these indels were consistent with those found in the genomes of *S. suis* T-I-64 and T-I-256. In the case of *S. suis* T-I-8, 24 of these indels were located within the CDS region, spanning across 20 different genes. These indels were associated with various biological processes, including metabolism, membrane protein synthesis, transposons, protein export, and cell wall surface anchor family proteins. However, 52 indels were found in *S. suis* T-I-64 and T-I-256 genomes. In addition to the same indels as *S. suis* T-I-8, these two strains displayed two unique indels within the CDS region, primarily linked to the purine salvage pathway and the cysteine pathway (Table 2).

**TABLE 1:** A summary of 11 SNPs in *S. suis^a^*^,^^*b*^

#CHROM	POS	POS	REF	T-I-256	T-I-64	T-I-8	Region	Type	Gene	Function
NC_012925.1	73309	73309	C	C	T	C	CDS	Nonsynonymous	gene-SSU_RS00435	50S ribosomal protein L4
NC_012925.1	255014	255014	A	C	A	A	CDS	Nonsynonymous	gene-SSU_RS01380	MIP/aquaporin family protein
NC_012925.1	697705	697705	T	T	T	C	Downstream and upstream	–	cds-WP_002935444.1-cds-WP_011922251.1	–
NC_012925.1	1123232	1123232	C	A	A	C	CDS	Nonsynonymous	gene-SSU_RS05600	50S ribosomal protein L20
NC_012925.1	1124391	1124391	T	C	T	T	Upstream and downstream	–	cds-WP_032497583.1-cds-WP_002937055.1	–
NC_012925.1	1221394	1221394	G	A	A	G	CDS	Nonsynonymous	gene-SSU_RS06070	Glycosyltransferase family 4 protein
NC_012925.1	1614291	1614291	A	C	C	A	CDS	Nonsynonymous	gene-SSU_RS08100	Acyl carrier protein
NC_012925.1	1640610	1640610	A	C	C	A	CDS	Nonsynonymous	gene-SSU_RS08200	Magnesium transporter CorA family protein
NC_012925.1	1849055	1849055	G	C	G	G	CDS	Stopgain	gene-SSU_RS09190	ABC transporter ATP-binding protein
NC_012925.1	1851356	1851356	C	A	A	C	CDS	Nonsynonymous	gene-SSU_RS09195	ABC transporter ATP-binding protein
NC_012925.1	1852443	1852443	T	G	G	T	CDS	Nonsynonymous	gene-SSU_RS09200	MarR family transcriptional regulator

^*a*^ –, not applicable.

^*b*^ CHROM, chromosome; POS, position; REF, reference strain.

**TABLE 2:** A summary of 52 indels in *S. suis*

#CHROM	POS	POS	REF	Type	T-I-256	T-I-64	T-I-8	Region	Type	Gene	Function
NC_012925.1	13100	13100	–	Insertion	1/1	1/1	0/0	CDS	Nonframeshift	gene-SSU_RS00070	Hypoxanthine phosphoribosyltransferase
NC_012925.1	71772	71772	A	Deletion	1/1	1/1	1/1	Downstream and upstream	–	cds-WP_014635810.1-cds-WP_011922546.1	–
NC_012925.1	87409	87415	TGCCAAA	Deletion	1/1	1/1	1/1	Downstream	–	cds-WP_012774898.1	–
NC_012925.1	87410	87413	GCCA	Deletion	1/1	1/1	1/1	Downstream	–	cds-WP_012774898.1	–
NC_012925.1	87420	87420	A	Deletion	1/1	1/1	1/1	Downstream	–	cds-WP_012774898.1	–
NC_012925.1	126107	126107	A	Deletion	1/1	1/1	1/1	Downstream and upstream	–	cds-WP_012775422.1-cds-WP_012774908.1	–
NC_012925.1	127988	127989	AA	Deletion	1/1	1/1	1/1	CDS	Frameshift	gene-SSU_RS00830	Folylpolyglutamate synthase/dihydrofolate synthase family protein
NC_012925.1	129155	129164	AAAATATAGA	Deletion	1/1	1/1	1/1	Upstream	–	cds-WP_011921730.1-cds-WP_012774909.1	–
NC_012925.1	146069	146069	A	Deletion	1/1	1/1	1/1	Downstream and upstream	–	cds-WP_011921748.1-cds-WP_011921749.1	–
NC_012925.1	163461	163469	CTTCTGAGC	Deletion	1/1	1/1	1/1	CDS	Nonframeshift	gene-SSU_RS00995	YSIRK-type signal peptide-containing protein
NC_012925.1	234833	234833	A	Deletion	1/1	1/1	1/1	CDS	Frameshift	gene-SSU_RS01275	DUF1430 domain-containing protein
NC_012925.1	259413	259424	GCAAACACCACA	Deletion	1/1	1/1	1/1	CDS	Nonframeshift	gene-SSU_RS01400	LPXTG cell wall anchor domain-containing protein
NC_012925.1	332301	332301	T	Deletion	1/1	1/1	1/1	Upstream	–	cds-WP_012774967.1	–
NC_012925.1	357506	357506	T	Deletion	1/1	1/1	1/1	CDS	Frameshift	gene-SSU_RS01860	Cysteine hydrolase family protein
NC_012925.1	396871	396871	T	Deletion	1/1	1/1	1/1	Downstream and upstream	–	cds-WP_004194171.1-cds-WP_004194167.1	–
NC_012925.1	396912	396913	GA	Deletion	1/1	1/1	1/1	Downstream and upstream	–	cds-WP_004194171.1-cds-WP_004194167.1	–
NC_012925.1	427004	427004	–	Insertion	1/1	1/1	1/1	Downstream	–	cds-SSU_RS10460	–
NC_012925.1	853630	853630	–	Insertion	1/1	1/1	1/1	Downstream and upstream	–	cds-WP_011922397.1-cds-SSU_RS10100	–
NC_012925.1	853881	853881	–	Insertion	1/1	1/1	1/1	CDS	Frameshift	gene-SSU_RS10100	Abi family protein
NC_012925.1	853947	853947	–	Insertion	1/1	1/1	1/1	CDS	Frameshift	gene-SSU_RS10100	Abi family protein
NC_012925.1	879988	879993	CTAGCT	Deletion	1/1	1/1	1/1	CDS	Nonframeshift	gene-SSU_RS04360	FAD-dependent oxidoreductase
NC_012925.1	910335	910337	CCC	Deletion	1/1	1/1	1/1	CDS	Nonframeshift	gene-SSU_RS04490	ZmpA/ZmpB/ZmpC family metallo-endopeptidase
NC_012925.1	967801	967801	T	Deletion	1/1	1/1	1/1	Upstream and downstream	–	cds-WP_012775157.1-cds-WP_012027071.1	–
NC_012925.1	1073309	1073309	T	Deletion	1/1	1/1	1/1	CDS	Frameshift	gene-SSU_RS10585	Hyaluronate lyase
NC_012925.1	1073790	1073791	AT	Deletion	1/1	1/1	1/1	CDS	Frameshift	gene-SSU_RS10585	Hyaluronate lyase
NC_012925.1	1084376	1084381	CAAAAT	Deletion	1/1	1/1	1/1	CDS	Nonframeshift	gene-SSU_RS05405	IS110 family transposase
NC_012925.1	1210965	1210968	GTGT	Deletion	1/1	1/1	1/1	Upstream and downstream	–	cds-WP_012027299.1-cds-WP_012027300.1	–
NC_012925.1	1220207	1220207	A	Deletion	1/1	1/1	1/1	CDS	Frameshift	gene-SSU_RS06065	Hypothetical protein
NC_012925.1	1246570	1246571	CA	Deletion	1/1	1/1	1/1	Upstream	–	cds-WP_012775226.1-cds-WP_012775227.1	–
NC_012925.1	1246634	1246634	–	Insertion	1/1	1/1	1/1	Upstream	–	cds-WP_012775226.1-cds-WP_012775227.1	–
NC_012925.1	1246643	1246644	AG	Deletion	1/1	1/1	1/1	Upstream	–	cds-WP_012775226.1-cds-WP_012775227.1	–
NC_012925.1	1246648	1246648	–	Insertion	1/1	1/1	1/1	Upstream	–	cds-WP_012775226.1-cds-WP_012775227.1	–
NC_012925.1	1246650	1246654	TAGAA	Deletion	1/1	1/1	1/1	Upstream	–	cds-WP_012775226.1-cds-WP_012775227.1	–
NC_012925.1	1251294	1251294	T	Deletion	1/1	1/1	1/1	CDS	Frameshift	gene-SSU_RS06175	Preprotein translocase subunit SecG
NC_012925.1	1252772	1252772	–	Insertion	1/1	1/1	1/1	CDS	Frameshift	gene-SSU_RS06190	Dephospho-CoA kinase
NC_012925.1	1252989	1252989	–	Insertion	1/1	1/1	1/1	CDS	Frameshift	gene-SSU_RS06190	Dephospho-CoA kinase
NC_012925.1	1252992	1252992	T	Deletion	1/1	1/1	1/1	CDS	Frameshift	gene-SSU_RS06190	Dephospho-CoA kinase
NC_012925.1	1262328	1262328	–	Insertion	1/1	1/1	1/1	CDS	Frameshift	gene-SSU_RS06235	HAD family hydrolase
NC_012925.1	1353156	1353156	–	Insertion	1/1	1/1	1/1	CDS	Frameshift	gene-SSU_RS10185	Hypothetical protein
NC_012925.1	1356965	1356965	G	Deletion	1/1	1/1	1/1	Upstream and downstream	–	cds-WP_162286914.1-cds-WP_012775258.1	–
NC_012925.1	1356972	1356972	–	Insertion	1/1	1/1	1/1	Upstream and downstream	–	cds-WP_162286914.1-cds-WP_012775258.1	–
NC_012925.1	1394635	1394635	–	Insertion	1/1	1/1	1/1	Upstream and downstream	–	cds-WP_012027489.1-cds-WP_012027490.1	–
NC_012925.1	1396137	1396138	TG	Deletion	1/1	1/1	1/1	Upstream and downstream	–	cds-WP_012027490.1-cds-WP_002937312.1	–
NC_012925.1	1396144	1396144	–	Insertion	1/1	1/1	1/1	Upstream and downstream	–	cds-WP_012027490.1-cds-WP_002937312.1	–
NC_012925.1	1470643	1470643	–	Insertion	1/1	1/1	1/1	CDS	Frameshift	gene-SSU_RS07350	IS110 family transposase
NC_012925.1	1492597	1492597	T	Deletion	1/1	1/1	1/1	CDS	Frameshift	gene-SSU_RS07455	YSIRK-type signal peptide-containing protein
NC_012925.1	1534545	1534547	AAT	Deletion	1/1	1/1	0/0	CDS	Nonframeshift	gene-SSU_RS07705	Endolytic transglycosylase MltG
NC_012925.1	1602665	1602667	CTT	Deletion	1/1	1/1	1/1	CDS	Nonframeshift	gene-SSU_RS08025	Restriction endonuclease subunit S
NC_012925.1	1677032	1677032	–	Insertion	1/1	1/1	1/1	Upstream and downstream	–	cds-WP_002942324.1-cds-WP_012775331.1	–
NC_012925.1	1811025	1811025	–	Insertion	1/1	1/1	1/1	CDS	Frameshift	gene-SSU_RS08975	3′−5′ exoribonuclease YhaM family protein
NC_012925.1	1916456	1916456	A	Deletion	1/1	1/1	1/1	CDS	Frameshift	gene-SSU_RS10595	Pilin N-terminal domain-containing protein
NC_012925.1	1958662	1958662	–	Insertion	1/1	1/1	1/1	Upstream and downstream	–	cds-WP_012028014.1-cds-WP_012028015.1	–

### Non-targeted high performance liquid chromatography-MS/MS metabolic profile analysis

**Fig 1:**
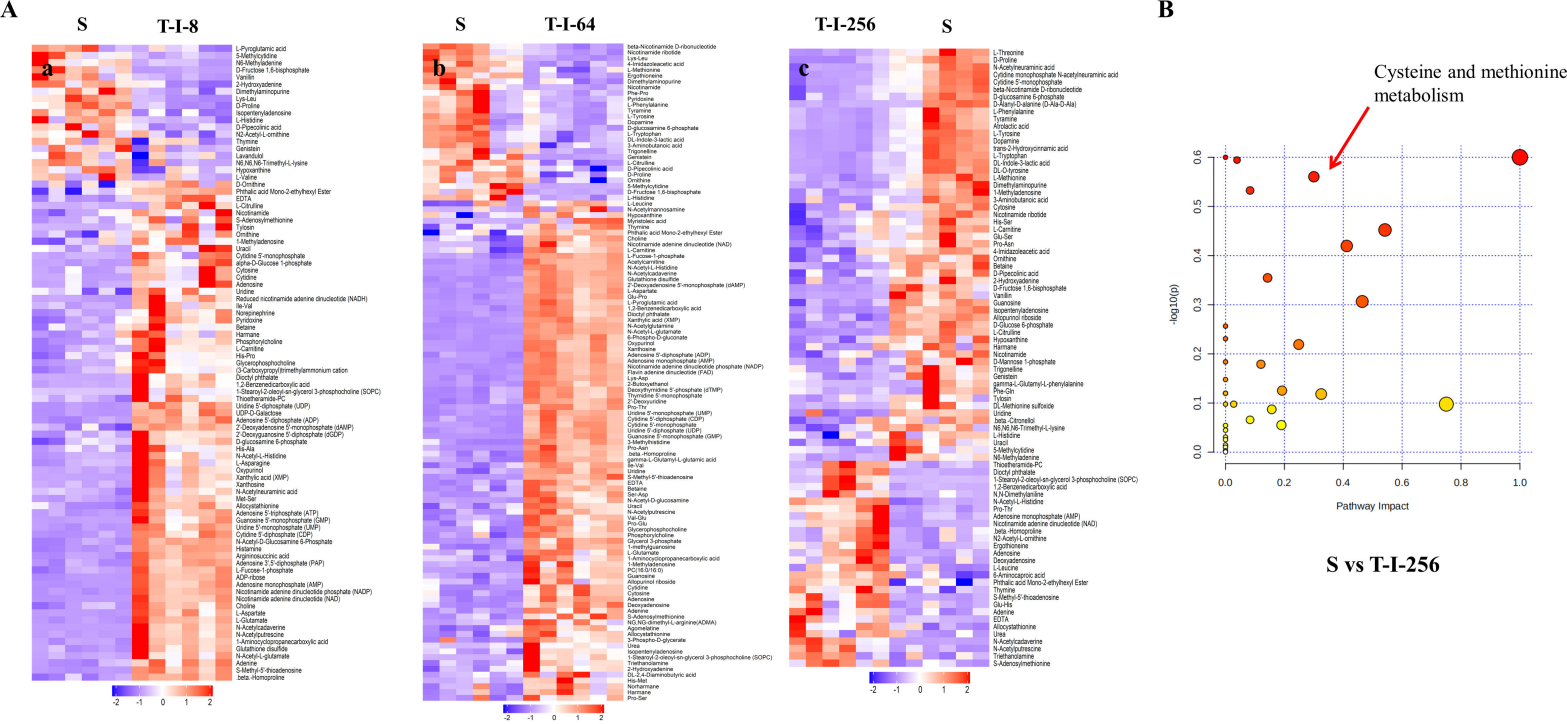
Bioinformatics analysis of the metabolomics of *S. suis*. (**A**) Overview metabolic profile heat map shows the comparison of control group S with different resistant strains in this study. The six biological replicates of the control group and the six biological replicates of the three resistant strain group are plotted on this heat map. (a) S vs T-I-8 group, (b) S vs T-I-64 group, and (c) S vs T-I-256 group. (**B**) Results of enrichment analysis of the KEGG pathway in the S and T-I-256 groups. The red arrows mark the cysteine and methionine metabolic pathways.

The metabolomic profiles of S and T-I-8, S and T-I-64, and S and T-I-256 were analyzed by liquid chromatography-mass spectrometry. The analytical performance was evaluated by injecting multiple quality control (QC) samples in between the biological samples, and the correlation coefficient of the QCs during the entire MS run was calculated as 0.98, which suggested that our analytical method is robust and reproducible. Each tested bacteria group also contained six biological replicates to ensure biological reproducibility. Unsupervised principal component analysis (PCA) and orthogonal partial least squares discriminant analysis (OPLS-DA) were used to evaluate the three groups of strains, and the results are shown in Table 3. The scores of R2Y and Q2 were both greater than 0.5, which proved that the model was established reliably and had good stability. To further decipher the metabolic differences between the three comparison groups, a combined comparison of metabolite *t*-test *P*-value and fold change (FC) of control samples compared to T-I-8, T-I-64, and T-I-256 samples was conducted, respectively. Differential metabolites were obtained by *t*-test (*P* < 0.05), fold change (FC > 1.5 or FC < 0.667), and model variable weight (variable influence of projection, VIP > 1) threshold screening. S and T-I-8, S and T-I-64, and S and T-I-256 groups were detected with 89, 113, and 84 differentially expressed metabolites, respectively (Fig. 1A; Tables S2 to S4). The pictures of differential metabolites are given in detail in the supplemental material (Fig. S1 to S3). Combining the results of the analysis of the three groups, it was found that the FC score of the differential metabolite L-met showed a regular decrease and was the lowest value in the S and T-I-256 groups (Table 4). Meanwhile, as shown in Fig. 1B; Table 5, we found that the metabolism of cysteine and methionine was significantly affected by the Kyoto Encyclopedia of Genes and Genomes (KEGG) pathway enrichment analysis in the S and T-I-256 groups. These results prompted us to explore whether the phenotype of *S. suis* changes in the presence of L-met.

**TABLE 3:** Statistical analysis of the metabolomics of the different porcine *S. suis* in this study^*a*^

Group	PCA	OPLS-DA	
	R2X (cum)	R2Y (cum)	Q2 (cum)
T-I-8 vs S	0.622	0.992	0.87
T-I-64 vs S	0.536	0.993	0.877
T-I-256 vs S	0.575	0.993	0.883

^*a*^ R2Y means that the square of the percentage of original data is retained in the *x*-axis direction; Q2 represents the prediction rate of the model.

**TABLE 4:** Changes in the metabolism of L-met in *S. suis* at different levels of drug resistance

Group	VIP	Fold change	*P*-value
T-I-256 vs S	1.789024472	0.478270847	0.000384802
T-I-64 vs S	1.591749366	0.473527031	0.004299033
T-I-8 vs S	0.52119909	0.990764742	0.969032166

**TABLE 5:** Results from pathway analysis^*a*^

	KEGG	Total	Expected	Hits	Raw *P*	−log_10_(*P*)	Holm adjust	FDR	Impact
1	Valine, leucine, and isoleucine biosynthesis	6	0.97	2	0.251	0.6	1	1	0
2	Inositol phosphate metabolism	6	0.97	2	0.251	0.6	1	1	1
3	Purine metabolism	49	7.93	10	0.254	0.594	1	1	0.04
4	Cysteine and methionine metabolism	22	3.56	5	0.275	0.561	1	1	0.3
5	Aminoacyl-tRNA biosynthesis	45	7.28	9	0.293	0.533	1	1	0.08
6	Glycerophospholipid metabolism	13	2.1	3	0.353	0.452	1	1	0.54
7	Arginine biosynthesis	8	1.29	2	0.381	0.42	1	1	0.41
8	Streptomycin biosynthesis	9	1.46	2	0.442	0.355	1	1	0.14
9	Methane metabolism	16	2.59	3	0.494	0.307	1	1	0.46
10	Valine, leucine, and isoleucine degradation	11	1.78	2	0.554	0.257	1	1	0

^*a*^ Total is the total number of compounds in the pathway. Hits refer to the actually matched number from the user-uploaded data. Raw *P* is the original *P* value calculated from the enrichment analysis. Holm *P* is the *P* value adjusted by Holm-Bonferroni method. FDR *P* is the *P* value adjusted using false discovery rate. Impact is the pathway impact value calculated from pathway topology analysis.

### Therapeutic effect of L-methionine combined with tylosin *in vivo*

**Fig 2:**
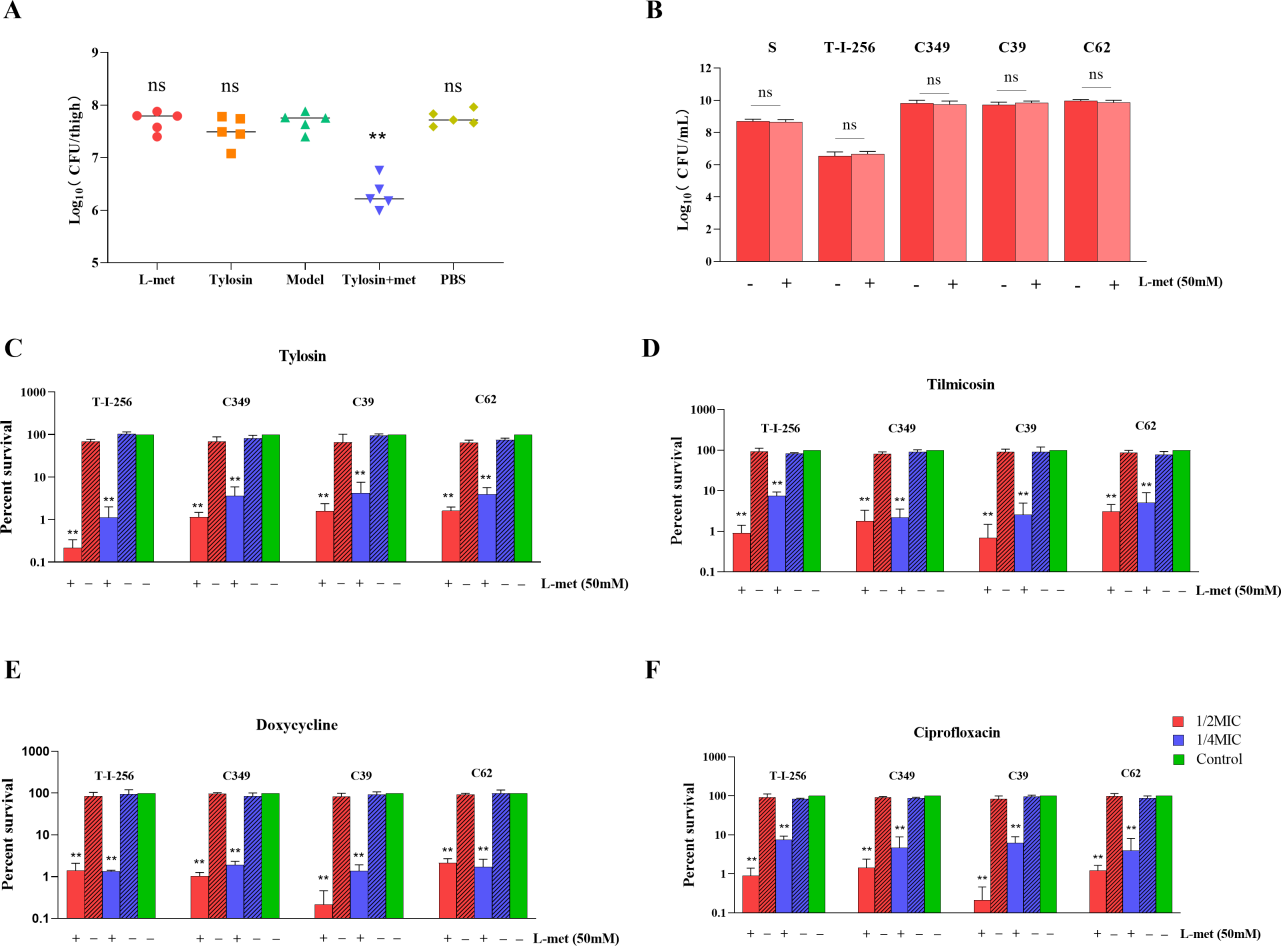
Effect of L-met addition on the survival of *S. suis* in *in vitro* culture and on the treatment of *S. suis* after host infection *in vivo*. (**A**) Therapeutic effects of tylosin and L-met alone and in combination in neutrophilic mice infected with drug-resistant strains. The bacterial load of infected thigh muscle in neutropenic mice (*n* = 5 per group) was determined by colony counting. Model: the mice with persistent 24 h infection after intramuscular injection of drug-resistant bacteria into the thigh. (**B**) Effect of adding 50 mM L-met on the survival of *S. suis*. (**C–F**) Synergistic effect of 50 mM L-met in combination with tylosin (**C**), tilmicosin (**D**), doxycycline (**E**), and ciprofloxacin. (**F**) Bacterial survival. Red, antibacterial drug concentration of 1/2 minimum inhibitory concentration (MIC) for strains; blue, antimicrobial drug concentration of 1/4 MIC for strains; green, control group without drugs. All data are the mean ± s.d. *P* values were determined using an unpaired, two-tailed Student’s *t*-test. ns, *P* > 0.05; *P < 0.05; and **P < 0.01.

A mouse thigh infection model was established to evaluate the synergetic effect of L-met and tylosin against drug-resistant strains of *S. suis in vivo*. The change in viable bacterial counts in the mouse thighs was determined by colony counting. The results showed that after the combined treatment with L-met and tylosin, the number of bacteria in the mouse thigh infection model was significantly reduced compared to that seen after treatment with either tylosin or L-met alone (Fig. 2A).

### L-methionine increased the susceptibility of planktonic *S. suis* cells to different drugs

To verify whether L-met was associated with resistance in *S. suis*, we used resistant strain T-I-256 obtained by laboratory induction and resistant strain C349, C39, and C62 obtained by clinical isolation, which were resistant to a variety of antimicrobial drugs and showed several times higher MIC (minimum inhibitory concentration) to tylosin, tilmicosin, doxycycline, and ciprofloxacin compared to *S. suis* S strain (Table 6). We also investigated the toxic effects of L-met on *S. suis*, and the results showed that the addition of 50 mM of L-met had no significant effect on the growth of either *S. suis* (Fig. 2B). We observed a significant decrease in bacterial survival after treatment with 1/2 MIC or 1/4 MIC drug levels of tylosin, tilmicosin, doxycycline, and ciprofloxacin in the presence of L-met (Fig. 2C through F). In particular, the combination of an antibacterial drug with 1/2 MIC and L-met achieved a bactericidal effect of more than 90% against resistant strains of *S. suis*, much higher than the bactericidal effect of the drug acting on *S. suis*. The time-kill growth curves also showed that the combination of 50 mM L-met with 1/2 MIC or 1/4 MIC concentration of tylosin can significantly inhibit the growth of bacteria (Fig. S4). These data suggest that L-met can enhance the susceptibility of resistant strains of *S. suis* to antimicrobial drugs.

**TABLE 6:** MIC (mg/L) for *S. suis* in this study

Strains	Tylosin	Tilmicosin	Ciprofloxacin	Doxycycline
*S. suis* S	0.125	0.25	0.0625	0.0625
*S. suis* T-I-256	256	16	2	4
*S. suis* C349	640	640	8	32
*S. suis* C39	640	640	16	32
*S. suis* C62	640	320	8	8

### L-methionine can affect methionine cycle metabolites

**Fig 3:**
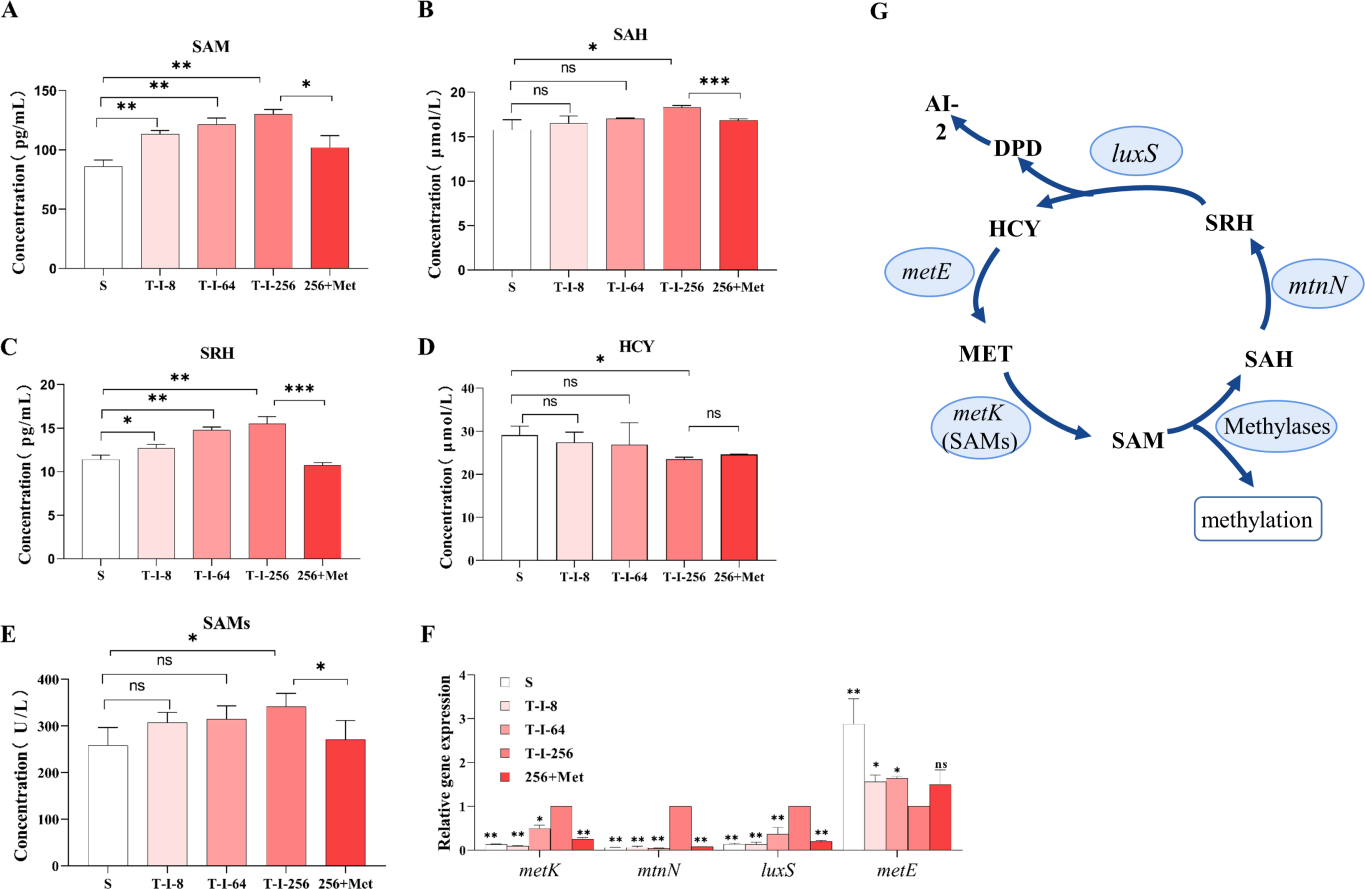
Metabolic pathway analysis. (**A–D**) SAM (**A**), SAH (**B**), SRH (**C**), and HCY (**D**) content of the MET metabolic pathway in S, T-I-8, T-I-64, T-I-256, and Met+256 strains. (**E**) SAMs activity of the MET metabolic pathway in S, T-I-8, T-I-64, T-I-256, and Met+256 strains. (**F**) The gene levels of the MET metabolic pathways in S, T-I-8, T-I-64, T-I-256, and Met+256 strains were examined by RT-PCR. All data are the mean ± s.d. *P* values were determined using an unpaired, two-tailed Student’s *t*-test. ns, *P* > 0.05; *P < 0.05; and **P < 0.01. (**G**) Schematic diagram of the MET metabolic pathway.

To understand how the methyl cycle is activated by L-met, we further assessed metabolite content, enzyme activity, and gene expression of the MET metabolic pathway. The concentrations of SAM, SAH, and S-ribose homocysteine (SRH) in *S. suis* were found to increase with the rising level of *S. suis* resistance, and HCY was found to decrease, and all the tested metabolic contents in the high-level resistant strain T-I-256 changed significantly compared with the sensitive strain *S. suis* (Fig. 3A through D). A significant increase in the metabolic contents of SAM, SAH, and SRH occurred after the T-I-256 strain was cultured in the medium that was spiked with L-met. SAMs are key enzymes that regulate the production of SAM from methionine. The results showed that the enzymatic activity of SAMs was upregulated with the increase of bacterial resistance (Fig. 3E). However, only the T-I-256 group was significantly different from the sensitive strain *S. suis*. Moreover, the activity of SAMs was significantly reduced when L-met was added exogenously to the T-I-256 strain. We analyzed the gene expression levels of *metK*, *mtnN*, *luxS*, and *metE* in the MET metabolic pathway, and the results showed that the gene expression levels of *metK*, *mtnN*, and *luxS* were significantly upregulated with increasing *S. suis* resistance, while *metE* was significantly downregulated in T-I-256 strains (Fig. 3F). When L-met was added exogenously to T-I-256, significant downregulation of *metK*, *mtnN*, and *luxS* expression occurred (Fig. 3F). This suggests that exogenous L-met can affect the MET metabolic pathway (Fig. 3G).

### L-methionine can affect methylation metabolism

**Fig 4:**
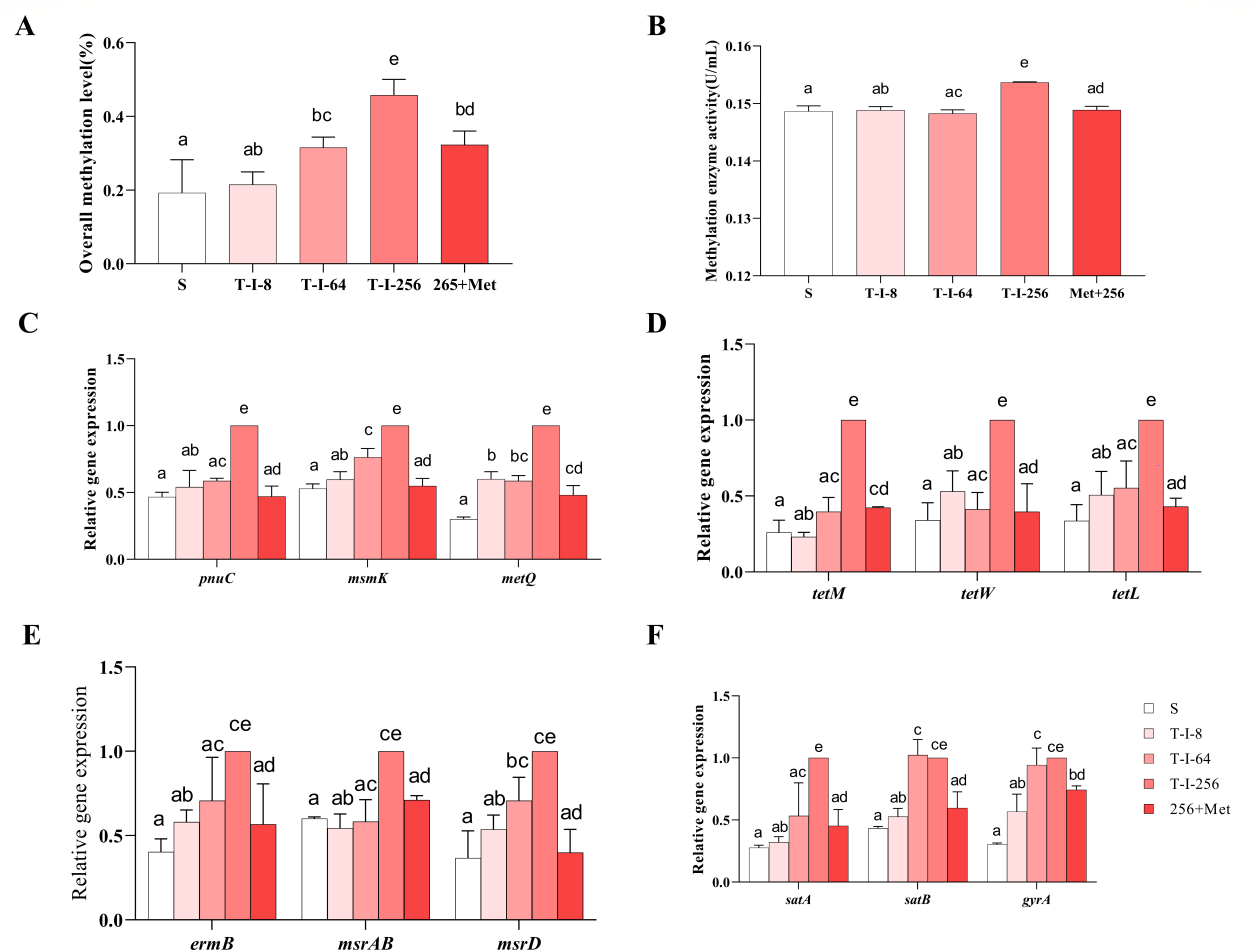
Effect of L-met addition on the methyl cycle of *S. suis*. (**A**) Total methylation levels of S, T-I-8, T-I-64, T-I-256, and Met+256 strains. (**B**) Methylesterase activity in S, T-I-8, T-I-64, T-I-256, and Met+256 strains. (**C**) Gene expression levels of regulatory membrane transporter proteins in S, T-I-8, T-I-64, T-I-256, and Met+256 strains. (**D–F**) Expression levels of tetracycline (**D**), macrolide (**E**), and fluoroquinolone (**D**) efflux pump genes in S, T-I-8, T-I-64, T-I-256, and Met+256 strains. The data are presented as means ± SEM. The significance of the differences was determined by one-way ANOVA. Bars that do not share the same letters are significantly different (*P <* 0.05) from each other.

MET is a synthetic precursor of the important methyl donor SAM, while SAM and SAH are important metabolites that affect the methyl cycle. The effect of L-met on SAM and SAH has been demonstrated. To further investigate the effect of L-met on the methyl cycle, we measured total methylation levels and methylation enzymes. The results showed that the total methylation level and methylation enzyme activity of *S. suis* increased significantly with the increase of drug resistance level (Fig. 4A and B). When L-met was added to T-I-256, the total methylation level and methylesterase activity were significantly decreased.

### Effect of L-methionine addition on drug transport

To determine whether the drug transport channels induced by the methyl cycle are affected by L-met, we analyzed the expression levels of genes regulating drug transport proteins and efflux pump genes in *S. suis* by RT-PCR. The expression of *pnuC* (nicotinamide riboside transporter) (24, 25), *msmK* (multiple sugar metabolism protein K, ABC transporter) (26, 27), and *metQ* (L-methionine/D-methionine ABC transporter membrane-anchored binding protein) (28, 29) genes, which regulate drug transport proteins, were significantly upregulated in strain T-I-256 compared with strain S (Fig. 4C). When L-met was exogenously added to strain T-I-256, the expression of *pnuC*, *msmK,* and *metQ* genes was significantly downregulated (Fig. 4C). We also examined the efflux pump genes of different drugs in *S. suis*. The results showed that the efflux pump genes *tetM*, *tetW*, and *tetL* of tetracyclines (Fig. 4D), *ermB*, *msrAB*, and *msrD* of macrolides (Fig. 4E), and *satA*, *satB*, and *gyrA* of fluoroquinolones (Fig. 4F) were significantly upregulated in strain T-I-256 compared to strain S. Moreover, the exogenous addition of L-met to the T-I-256 strain significantly suppressed the efflux pump genes.

### Effect of L-methionine addition on oxidative stress

**Fig 5:**
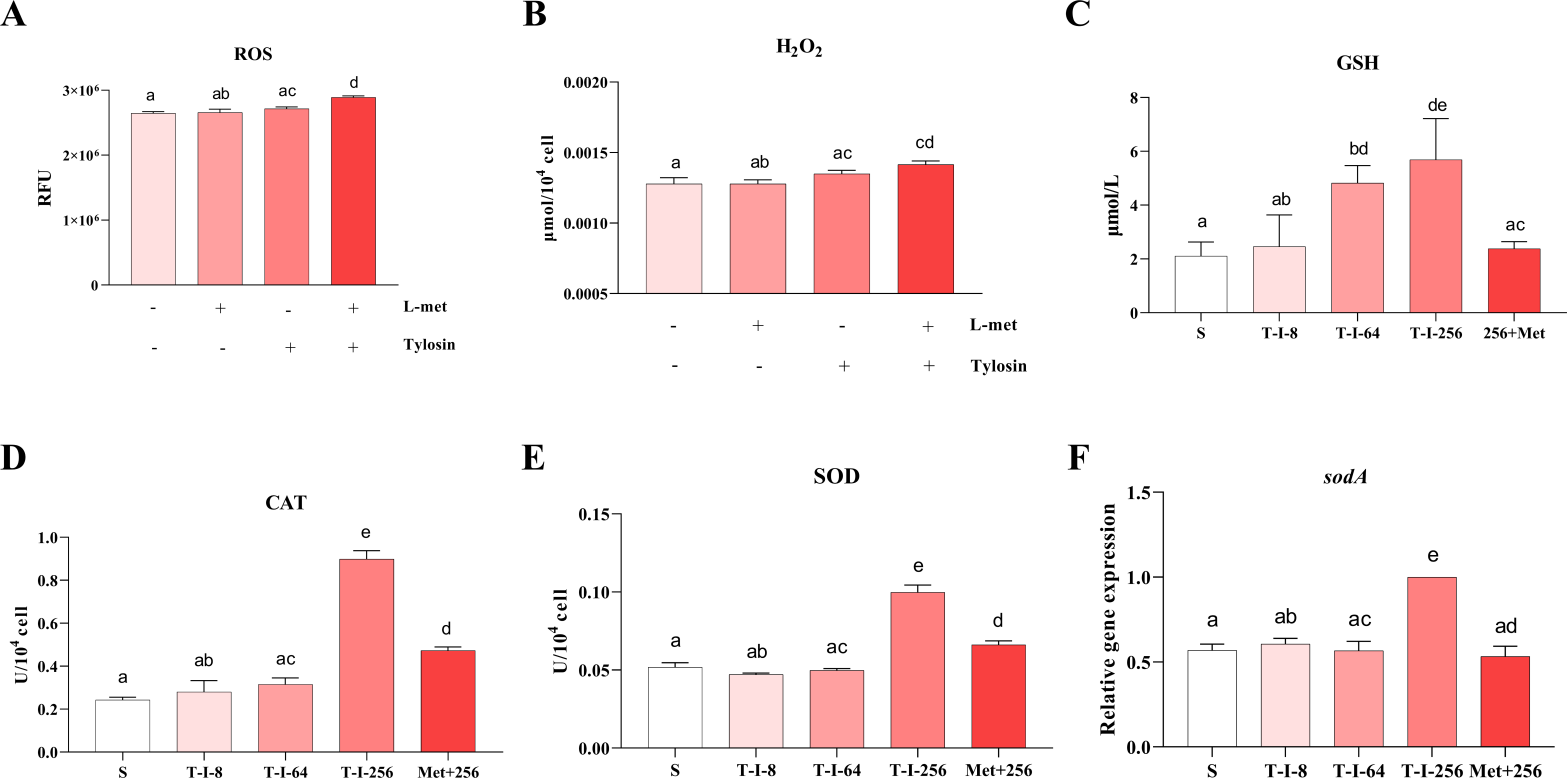
Effect of L-met addition on the level of oxidative stress in *S. suis*. (**A and B**) Effect of 50 mM L-met in combination with tylosin on ROS (**A**) and H_2_O_2_ (**B**) concentration in T-I-256 strains of *S. suis*. (**C**) GSH content in S, T-I-8, T-I-64, T-I-256, and Met+256 strains. (**D and E**) CAT (**D**) and SOD (**E**) activities in S, T-I-8, T-I-64, T-I-256, and Met+256 strains. (**F**) Expression levels of *sodA* genes in S, T-I-8, T-I-64, T-I-256, and Met+256 strains. The data are presented as means ± SEM. The significance of the differences was determined by one-way ANOVA. Bars that do not share the same letters are significantly different (*P <* 0.05) from each other.

To determine the effects of oxidative stress caused by altered methyl cycle, we measured ROS levels, glutathione (GSH) content, oxidative stress enzyme activity, and *sodA* gene (superoxide dismutase SodA) expression (30, 31). ROS levels were compared after the addition of macrolides alone or in combination with L-met, using DCFH-DA. The results showed that there was a significant increase in ROS levels within *S. suis* after the combination of tylosin and L-met (Fig. 5A). It was also found that the H_2_O_2_ level in *S. suis* increased significantly after the combination of tylosin and L-met (Fig. 5B). The level of GSH was also found to increase significantly with increasing levels of *S. suis* resistance (Fig. 5C). In contrast, the exogenous addition of L-met to strain T-I-256 significantly reduced the level of GSH in the altered strain (Fig. 5C). Also, the scavenging of ROS by superoxide dismutase (SOD) and catalase (CAT) was significantly enhanced in strain T-I-256 (Fig. 5D and E). In contrast, both CAT and SOD activities and *sodA* gene were significantly decreased when supplemented with L-met (Fig. 5D through F).

### L-methionine affects metal starvation in *S. suis*

**Fig 6:**
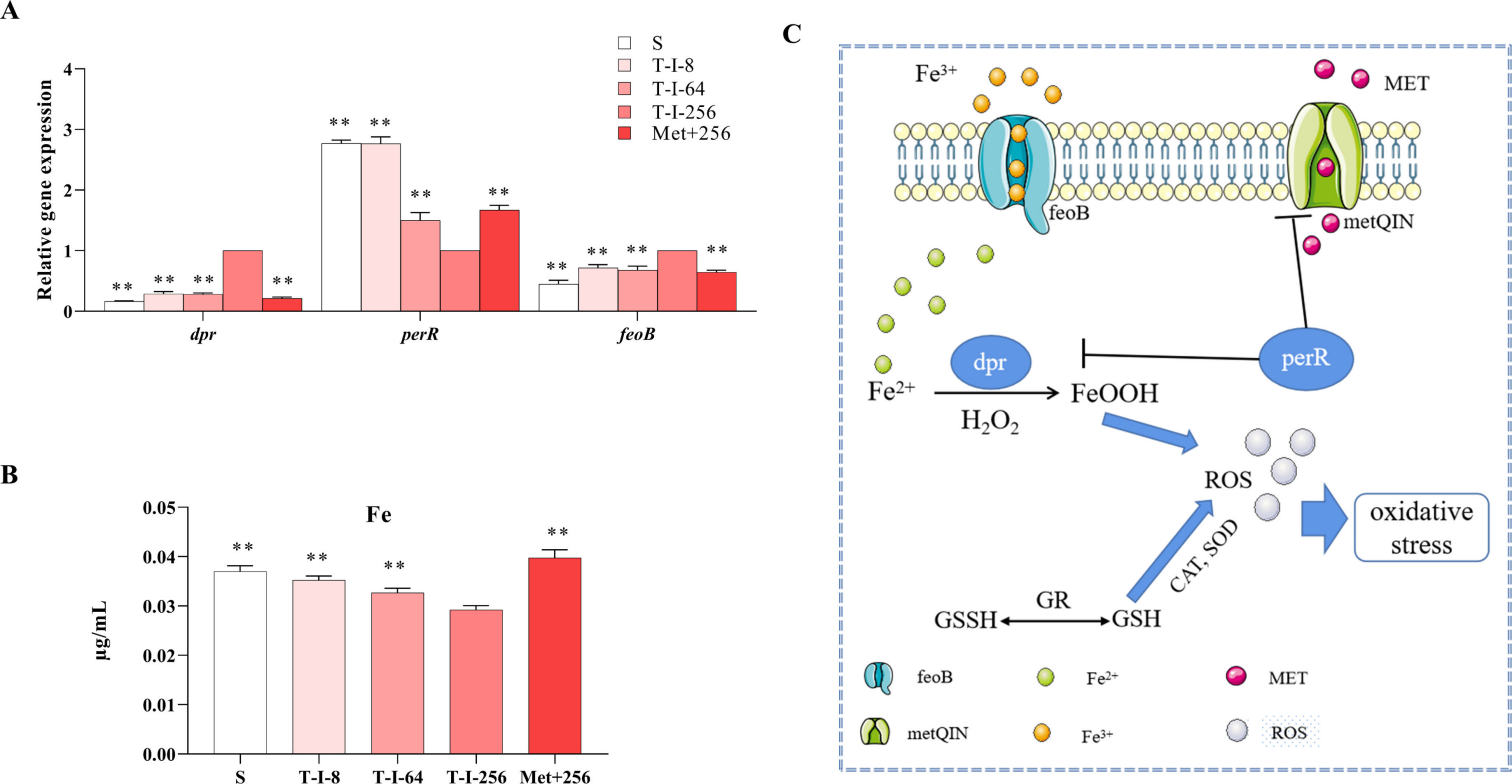
Effect of L-met addition on iron metal starvation in *S. suis*. (**A**) Expression levels of iron metal starvation-related genes in S, T-I-8, T-I-64, T-I-256, and Met+256 strains. (**B**) S, T-I-8, T-I-64, T-I-256, and Met+256 strains for the concentration of iron. The data are presented as means ± SEM. The significance of the differences was determined by one-way ANOVA. Bars that do not share the same letters are significantly different (*P <* 0.05) from each other. (**C**) Mechanism of action of MET in porcine *S. suis* on the effects of iron metal starvation and oxidative stress.

To understand whether the altered survival of *S. suis* by L-met is related to the modulation of the metal starvation response by fur-like proteins, the fur-like protein regulating gene *perR* was determined to be significantly downregulated in strain T-I-256, which reduced the repressive effect of *perR* on *dpr* (*dps*-like peroxide resistance protein) and caused a significant upregulation of gene expression of *dpr* (Fig. 6A). Gene expression of *dpr* significantly decreased and *perR* significantly increased after the exogenous addition of L-met in strain T-I-256 (Fig. 6A). The repression of *dpr* gene contributed to the Fenton response. Therefore, we determined the expression of the *feoB* gene (ferrous iron transport protein B), which regulates the iron transport protein, and the concentration of iron (32, 33). The results showed that the expression of *feoB* gene in T-I-256 strain was significantly upregulated, which promoted the efflux of iron and reduced the concentration of iron in the bacteria (Fig. 6A). Moreover, the expression of *feoB* gene was suppressed after the exogenous addition of L-met in strain T-I-256, which contributed to the accumulation of iron ions and promoted the Fenton reaction (Fig. 6A and B). The above results suggest that the effect of L-met addition on the survival of *S. suis* may be related to metal starvation (Fig. 6C).

## DISCUSSION

*S. suis* causes systemic infectivity in pigs and is one of the main causes of morbidity and mortality in piglets (34). However, the emergence of multidrug resistant strains has made the prevention, control, and treatment of bacterial diseases more difficult in clinical practice (35). Studies have shown that various endogenous metabolites, such as indole, NO, H_2_S, and amino acids, are associated with the development of bacterial drug resistance (36–38). These amino acids are the building blocks of proteins and precursors of functional molecules (39, 40). They are indispensable in certain developmental and physiological situations and are essential for normal physiology. Clinically, some essential amino acids are often used as supplements to reduce damage from pathogenic microorganisms and improve growth performance (41). The exogenous addition of citrulline and glutamine can also enhance the susceptibility of *Salmonella* to the drug in *in vitro* cultures by modulating the TCA cycle to cause enhanced proton drive, leading to the accumulation of apramycin in the bacteria (42). This demonstrates that the physiological metabolic state of bacteria is altered when they develop drug resistance and that the exogenous addition of metabolites, by modulating metabolic pathways, allows bacterial resistance to be affected.

We conducted a resequencing analysis of *S. suis* with different levels of resistance induced by tylosin (*S. suis* T-I-8, T-I-64, and T-I-256). The results indicated that the SNPs in *S. suis* T-I-64 and T-I-256 were highly identical, and the indels in all three strains were also highly consistent. These findings suggest that the genetic backgrounds of *S. suis* with varying tylosin resistance levels induced by tylosin are highly identical, especially in *S. suis* T-I-64 and T-I-256. Furthermore, mutations in genes related to tylosin resistance mechanisms were not observed. Therefore, metabolomics can be employed to further investigate the reasons behind the development of resistance. In order to investigate the mechanism of drug resistance development in *S. suis* and to find countermeasures, we performed metabolomic assays using *S. suis* with different levels of drug resistance separately from standard strains to analyze physiological metabolic differences. We found that the inhibition of L-met synthesis by strains T-I-8, T-I-64, and T-I-256 gradually increased, and the L-met content decreased significantly. MET has been reported to exert effects on metabolism, oxidative stress, and disease development. Restriction of MET prevents alterations in transmethylation metabolism, thereby reducing DNA damage and pathogenic processes (43). MET restriction stimulates glutathione production while decreasing oxidative stress (44). At the same time, when MET intake increases, the flux of substrates through the transmethylation pathway decreases and the flux through the transsulfuration pathway increases (45). This explains well the metabolic changes that occur after the development of drug resistance in *S. suis*. The emergence of drug resistance has led to the limitation of methionine, which implies that the methyl metabolism is disturbed and the resistance of GSH to oxidative stress is enhanced (46). Significant upregulation of gene expression of exocytosis pumps and membrane transport proteins was also detected in drug-resistant strains. This demonstrates that the development of drug resistance reduces the intracellular accumulation of the drug, resulting in weakening of the inhibitory effect of the drug.

Peng et al. (47) found that exogenous alanine or glucose restores the susceptibility of multidrug-resistant *Edwardsiella tarda* to killing by kanamycin, demonstrating an approach to killing multidrug-resistant bacteria. Therefore, we refer to the method of Peng et al. (47) to restore the metabolic disorders and drug resistance caused by MET restriction by the exogenous addition of MET assay. First, we determined the effect of exogenous L-met addition on the resistance of *S. suis*. In *in vitro* tests, the bactericidal efficacy of tylosin, tilmicosin, ciprofloxacin, and doxycycline against resistant strains was significantly enhanced by the addition of exogenous L-met. It was also found in *in vivo* experiments that the combination of L-met with tylosin significantly improved the therapeutic effect of tylosin in mice infected with *S. suis*. This demonstrates that drug-resistant strains can take up MET from the medium to recover from the effects of MET restriction.

Our experiments also showed that the addition of L-met led to a decrease in SAM concentration, which caused a disturbance in methyl metabolism and resulted in the suppression of total methylation levels. The metabolite content, enzyme activity, and gene expression were measured in resistant strains after the exogenous addition of L-met to elucidate the relationship between resistance development and MET metabolism in *S. suis*. The results showed that the concentrations of SAH, SAM, and SRH, the gene expression of *metK*, *luxS*, *mtnN,* and *metE*, and the activity of SAMs were significantly altered in the resistant strains after the addition of L-met. We, therefore, hypothesize that the decrease in methyl donors due to the reduction in SAM concentration restores the metabolic level of resistant bacteria to a level similar to that of sensitive strains. The inhibition of methylation levels and methylesterase activity also contributed to the recovery of susceptibility of the resistant strains (48). It has been reported that methylation of the methylesterase Cfr, which reduces the ability of antibacterial drugs to bind to bacteria, leads to enhanced resistance of bacteria to chloramphenicol, lincomycin, and some macrolides (49). Methylation of Erm methylesterase can also cause the development of bacterial resistance to macrolides and lincosamides, among others (50). All these research demonstrate the importance of the effect of altered methylation on bacterial drug resistance.

The recovery of susceptibility of *S. suis* after L-met addition may also be a result of methylation affecting the transmembrane transport of the drug. Methyltransferases can impair transmembrane transport-mediated drug resistance in *Escherichia coli* by affecting the expression of the *sugE* gene (19). In our study, we also found that the addition of L-met significantly downregulated the expression of the *metQ* gene, which regulates membrane transport in drug-resistant strains (28, 29), and the expression of efflux pump genes of several drugs in drug-resistant bacteria was significantly downregulated by the action of L-met, which implies that the addition of L-met may contribute to the accumulation of the drug.

In addition, the effect of MET on oxidative stress and *PerR* fur-like protein-regulated metal starvation during *S. suis* infection cannot be ignored (51). The *perR* gene was found to significantly reduce the susceptibility of *S. suis* to H_2_O_2_ after its deletion (52). We also found that the gene *perR* was significantly upregulated in drug-resistant strains after L-met addition, whereas the *dpr* gene was significantly downregulated in drug-resistant strains after L-met addition. The inhibition of *dpr* allows the Fenton reaction to occur normally (53). Thus, when L-met was added, the level of ROS under the effect of tylosin was significantly increased, contributing to ROS-induced oxidative stress, which allows toxic hydroxyl radicals to be generated (54). The gene *sodA* can encode protein superoxide dismutase SodA. The main function of SodA is scavenging ROS (30, 31). The detection of diminished SOD and CAT activities and downregulation of *sodA* gene expression also confirmed the suppression of protective effects against oxidative stress in drug-resistant bacteria (55).

Overall, these results suggest that the MET metabolic pathway is associated with the development of drug resistance in *S. suis*. L-met may influence the development of resistance in *S. suis* through methyl metabolism and metal starvation. This study provides a new perspective on the mitigation of drug resistance in *S. suis*.

## MATERIALS AND METHODS

### Strains and growth conditions

The *S. suis* ATCC 700794 used in this study was purchased from the American Type Culture Collection (ATCC) and kept in our laboratory. Unless otherwise stated, bacteria were cultured in Todd-Hewitt Broth (THB) at 37°C under aeration. *S. suis* strain ATCC 700494 with different levels of resistance to macrolides, named T-I-8 (MIC 8 mg/L), T-I-64 (MIC 64 mg/L), and T-I-256 (MIC 256 mg/L), was obtained using *in vitro* laboratory induction under long-term selection pressure of tylosin (56). *S. suis* ATCC 700794 was grown in 96-well plates with increased tylosin concentrations (1/8–4 MIC). The inoculated microplates were incubated at 37℃ for 24 h before being evaluated. The strains surviving in the highest drug concentration were transferred to new 96-well plates with increasing drug concentrations. The bacteria continued to pass through generations until strains with different tylosin resistance levels were induced. In addition, the stability of tylosin resistance was tested by serial passage (10 times) on an antibiotic-free medium. The standard strain ATCC 700494 was described by the abbreviation S. In addition, we also selected three clinically isolated strains of *S. suis*: C349, C39, and C62, which were used in this study. All experiments were performed using bacteria in the exponential phase. We used the minimum inhibitory concentration to assess the sensitivity to antibiotics.

### Antibiotics and chemicals

Tylosin, tilmicosin, doxycycline, ciprofloxacin, and L-methionine (L-met) were purchased from Solarbio (Beijing, China). L-met was prepared with ddH_2_O amino acid stock solution (500 mM). After filtering the stock solution with a sterile hydrophilic PVDF with a membrane pore size of 0.22 µm (Biosharp, China), it was stored at –20°C. The drugs were diluted to the required concentration.

### MIC determination

All isolates were subjected to antimicrobial susceptibility testing via broth microdilution according to the procedure described by the Clinical Laboratory Standards Institute (57). In brief, tylosin, tilmicosin, doxycycline, or ciprofloxacin were mixed with an equal volume of bacterial broth containing 1.5 × 10^6^ CFUs mL^−1^ and twofold diluted in clear 96-well microtiter plates (Corning, NY, USA). The lowest concentration of the drugs at which no bacterial growth was observed after 24 h of incubation at 37°C was defined as the MIC (58). All samples were tested at least three times for MIC.

### Whole-genome resequencing for *S. suis* strains

Total DNA was extracted from *S. suis* using the E.Z.N.A. Tissue DNA kit (Omega Bio-Tek) following the Tissue DNA-Spin Protocol. After the DNA samples were delivered, a quality control test was carried out on the specimens, and the qualified DNA (>3 µg; concentration > 30 ng/µL; OD_260_/OD_280_ = 1.80–2.00) was used for further study. The genomic DNA library was constructed using the Illumina pair-end sequencing method and selected with an average insert size of ~450 bp using Illumina’s standard genomic DNA library preparation procedure. Sequencing of T-I-8, T-I-64, and T-I-256 was performed with the Illumina NovaSeq 6000 platform (150 bp × 2, Shanghai Biozeron Biotechnology Co., Ltd, Shanghai, China). The public data of reference genome of *Streptococcus suis* P1/7 were obtained from the NCBI (https://www.ncbi.nlm.nih.gov/nuccore/NC_012925.1/). The gene annotation information (including functional annotation and gene family information) was also downloaded in the corresponding genome database. The clean sequencing reads are aligned to the reference genome sequence using BWA (http://bio-bwa.sourceforge.net/) software (bwa mem -k 32). The valid BAM file was used to detect SNPs and short InDel by GATK (version 4.1.2.0) “HaplotypeCaller” function (http://www.broadinstitute.org/gatk/). When the gene annotation file of reference genome is provided, the annotation of detected variations can be performed by ANNOVAR (http://www.openbioinformatics.org/annovar/), including SNP (synonymous or non-synonymous mutations of SNPs) and InDel. Structure variations were identified by BreakDancer (http://breakdancer.sourceforge.net).

### Metabolome assay and data analysis

For targeted metabolomics analysis, the samples were taken out at −80°C, and 1mL of methanol acetonitrile aqueous solution (2:2:1, vol/vol) was added. The vortex was for 60 s, and ultrasound was performed twice at low temperatures for 30 min. The samples were placed at −20°C for 1 h to precipitate the protein and centrifuged at 14,000 rpm at 4°C for 20 min, and the supernatant was freeze-dried for subsequent tests. The samples were analyzed by ultra high performance liquid chromatography (1290 Infinity LC, Agilent Technologies) and coupled quadrupole time-of-flight (AB Sciex TripleTOF6600) equipped with an electrospray ionization source. Separation was achieved by a hydrophilic interaction chromatography on an ACQUITY UPLC BEH Amide column [2.1 × 100 mm, 1.7 µm particle size (Waters, Ireland)] using a gradient of solvent A (25 mM ammonium acetate and 25 mM ammonium hydroxide in water) and solvent B (acetonitrile). The gradient was 95% B for 0.5 min, linearly reduced to 65% in 7 min, and then reduced to 40% in 8 min, kept for 2 min, and then increased to 95% in 9.1 min, with a 4 min re-equilibration period employed. The flow rate was 0.3 mL/min, column temperature was 25°C, autosampler temperature was 4°C, and injection volume was 2 µL. Each sample was detected in both negative and positive ion modes. The raw MS data were converted into the MzXML files using a ProteoWizard MSConvert tool (59) and processed using the XCMS (60) for feature detection, retention time correction, and alignment. The metabolites were identified by accuracy mass (<25 ppm) and MS/MS data, which matched with our standard database. For multivariate statistical analysis, the web-based MetaboAnalyst system (https://www.metaboanalyst.ca/) was used, followed by the Pareto-scaling, PCA, and OPLS-DA. The significantly different metabolites were determined based on the combination of a statistically significant threshold of variable influence of projection value obtained from the OPLS-DA model and two-tailed Student’s *t*-test (*P*-value) on the raw data, and the metabolites with VIP values larger than 1.0 and *P*-values less than 0.05 were considered as significant.

### Thigh infection model experiment in mice

A mouse thigh infection model (61) was used to assess the synergistic effect of the combination of L-met and tylosin on *S. suis*. Six-week-old female specific pathogen-free ICR mice were purchased from the Experimental Animal Center of the Second Affiliated Hospital of Harbin Medical University (Harbin, China).The mice developed neutropenia (neutrophil count < 100 mm^3^) when cyclophosphamide was administered intraperitoneally at doses of 150 and 100 mg/kg of body weight, respectively, 4 days and 1 day before bacterial inoculation. The immunodeficient mice were divided into five groups for subsequent experiments (*n* = 5). Mice in the model group were injected intramuscularly with 100 µL of inoculum in each thigh, including 10^7^ log-phase test strains. Two hours after modeling, mice were treated with tylosin (10 mg/kg of body weight) and L-met (0.1 g/kg of body weight) alone or in combination as the tylosin single treatment group, L-met single treatment group, and combination treatment group, respectively. Finally, the mice injected with physiological salt were used as blank controls. Both L-met and tylosin were administered intramuscularly. After 22 h of drug treatment, mice were euthanized. Both posterior thigh muscles of the mice were immediately collected in normal saline, homogenized, and appropriately diluted, and the number of CFUs was determined by planar colony counting. The thigh tissue CFU titer is indicated as log_10_ CFU/thigh.

### Antibiotic survival assay

To obtain exponential and mid-stationary-phase cultures in THB, the 16-h culture was diluted 1:1,000 in fresh medium and grown to the desired turbidity (OD_600_ = 0.5). The sample was washed twice with PBS, and the culture was collected by centrifugation at 8,000 *g* for 8 min, and then resuspended in THB medium. Different antibiotics (tylosin, tilmicosin, doxycycline, and ciprofloxacin) with concentrations of 1/2 and 1/4 MIC were added, respectively. For the amino acid supplement experiment, L-met was added to the exponential phase culture at the specified concentration. After incubation, 100 µL aliquots were periodically taken out to perform serial dilutions and then inoculated (10 µL aliquots) on THB agar plates. The agar plates were incubated at 37°C for 24 h, and the number of cfu was calculated. Bacterial survival was calculated as the ratio between the dosing and blank control groups. The results represented the average of three biological replicates, and the error bars represent the standard deviation.

### Metabolite concentration detection

MET, SAM, SAH, and HCY concentrations were detected through MET, SAM, SAH, and HCY ELISA Kits, respectively (Sinobestbio, China). GSH level was determined using a content assay kit (Nanjing Jiancheng Bioengineering Institute, China). T-I-256 and L-met were incubated at 37°C for 12 h and named Met+256. The S, Met + 256, T-I-8, T-I-64, and T-I-256 cultures were collected, washed, resuspended in PBS, and adjusted to OD_600_ of 0.5. After sonicating and centrifuging 1 mL of the sample to break up the cells, the supernatant was collected and analyzed according to the kit to detect the concentration of metabolites in the bacteria. The assays were repeated three times.

### Measurement of enzyme activity

The activities of SAMs and methylases were detected by SAMs and methylases ELISA kit (Sinobestbio, China). The S, Met+256, T-I-8, T-I-64, and T-I-256 cultures were collected, washed, resuspended in PBS, and adjusted to OD_600_ of 0.5. After centrifugation, the supernatant was transferred to a new tube and reacted according to the manufacturer’s instructions, and absorbance at 450 nm was measured using the microplate reader. The activity of the corresponding enzyme was calculated by creating a standard curve and substituting the absorbance value of the sample.

SOD and CAT levels were measured using colorimetric assay kits (Nanjing Jiancheng Bioengineering Institute, China). In brief, bacterial cultures were collected, washed, resuspended in lysis buffer (from the assay kits), and disrupted by sonication. After centrifugation, the supernatant was transferred to a new tube and reacted according to the manufacturer’s instructions, and absorbance at 560 and 240 nm was measured using the microplate reader. SOD and CAT levels were quantified using a commercially available colorimetrical kit (62). The activity of the corresponding enzyme was calculated by creating a standard curve and substituting the absorbance value of the sample.

### Quantification of metabolism-related genes by RT-PCR

RT-PCR was used to analyze the relative expression of genes in this study. The genes *metK*, *mtnN*, *luxS*, and *metE* in the methionine pathway were detected. In order to explore whether exogenous methionine affects the efflux pump of *S. suis*, the genes *pnuC*, *msmK*, and *metQ* were detected. Drug resistance genes and efflux pump genes *tetM*, *tetW*, and *tetL* of tetracyclines, *ermB*, *msrAB*, and *msrD* of macrolides, *satA*, *satB,* and *gyrA* of fluoroquinolones were detected. Also, the genes *sodA* (superoxide dismutase SodA), the fur-like protein regulating gene *perR, dpr* (*dps*-like peroxide resistance protein), and *feoB* (ferrous iron transport protein B) associated with oxidative stress response and metal starvation were detected. The primer sequences used are listed in Table S1. The bacterial cultures of S, Met+256, T-I-8, T-I-64, and T-I-256 were collected by centrifugation. Total RNA extraction (Omega, China) and cDNA synthesis (Takara, China) were done according to the manufacturer’s instructions. The relative expression was normalized using the *16S rRNA* gene as an endogenous control (56). The reaction conditions were 94°C for 10 min followed by 40 cycles of amplification at 94°C for 15 s and 60°C for 60 s (63). Three replicate analyses were performed for each sample, and expression data were collected from three biological replicate samples. All assays were performed in triplicates.

### Total methylation level determination

DNA was extracted from strains S, T-I-8, T-I-64, T-I-256, and T-I-256 treated with L-met using a bacterial genomic DNA extraction kit (A&D Technology Corporation, China). Global DNA methylation was determined with the MethylFlash Methylated DNA 5-mC Quantification kit (Colorimetric) (EpiGentek, Farmingdale, New York, NY, USA). The level of methylation in DNA was then detected using the following colorimetric assay. The extracted DNA was added to the binding solution (provided by the manufacturer) to bond the DNA to the microplate, incubated at 37°C for 90 min, and then eluted with deionized water. A capture antibody for methylated DNA was added, sealed at indoor temperature, and incubated for 30 min before elution. After the addition of the detection antibody as well as the enhancement solution (provided by the manufacturer), it was incubated for 10 min at room temperature and protected from light. At the same time, the color change in the sample wells vs the positive control wells was monitored as sufficiently methylated DNA turned blue. The reaction of the enzyme was terminated by the addition of a fluorogenic developer, and the absorbance was measured and read at 450 nm using a fluorescence analyzer.

### Determination of iron concentrations

Cellular iron levels were determined using the colorimetric ferrozine assay method described by Riemer et al. (64). Cultures of *S. suis* were incubated to OD_600_ = 0.5, after the addition of L-met, and the cultures were incubated for 12 h and harvested by centrifugation. The cell pellet was washed twice with ice-cold PBS and resuspended in 1 mL of 50 mM NaOH. The lysate was mixed with 10 mM HCl and an iron release reagent and incubated at 60°C for 2 h to quantify the bound iron. After the mixture was cooled to 25°C, 30 mL of the iron detection reagent (6.5 mM ferrozine, 6.5 mM neocuproine, 2.5 M ammonium acetate, and 1M ascorbic acid dissolved in water) was added. After 30 min, the absorbance of the samples was measured at 550 nm.

### Measurement of oxidative stress indicators

ROS were detected by the Reactive Oxygen Species Assay Kit based on 2′,7′-dichlorodihydrofluorescein diacetate (DCFH-DA) (Beyotime Biotechnology, China). The samples were incubated with 10 mM DCFH-DA for 20 min. After washing by PBS, cells were treated with tylosin in the presence or absence of 50 mM L-met. Samples were taken after incubated for 20 min and used to monitor fluorescence intensity at an excitation wavelength of 488 nm and an emission wavelength of 525 nm.

GSH and H_2_O_2_ (Nanjing Jiancheng Bioengineering Institute, China) levels were determined using content assay kits (Nanjing Jiancheng Institute of Biological Engineering, Nanjing, Jiangsu, China). The resistant strain was incubated in THB medium supplemented with L-met for 12 h at 37°C, and the bacterial cultures were collected, washed, resuspended in PBS, and adjusted to an OD_600_ of 0.5. After sonicating and centrifuging 1 mL of the sample to break up the cells, the supernatant was collected and analyzed according to the kit instructions to detect the concentration of metabolites in the bacteria. The assays were repeated three times.

### Statistical analysis

Data were collected for statistical analysis, and Student’s *t*-test or one-way ANOVA was conducted using GraphPad Prism 8 (GraphPad Software, Inc., San Diego, CA, USA) and SPSS version 26.0 software (IBM SPSS, Chicago, IL, USA, software version 26.0) (65). Values were reported as the mean ± standard deviation. The different levels of statistical significance were set as ns, *P* > 0.05, *P < 0.05, and **P < 0.01.

## Data Availability

The data that support the findings of this study are available from the corresponding author upon reasonable request. Additionally, raw metabonomic data can be viewed in NODE (https://www.biosino.org/node) under accession no. OEP004815. Resequencing data are available under accession number PRJNA1052407.

## SUPPLEMENTAL MATERIAL

Tables S1 to S4 spectrum.02803-23-s0001.docx Primers used for the quantitative RT-PCR analysis and differential metabolites.

Fig. S1 to S4 spectrum.02803-23-s0002.pdf Fig S1 to S3 (Bioinformatics analysis of the metabolomics of *S. suis*) and Fig S4 (The time-kill curves of *Streptococcus suis* treated with tylosin and methionine at different concentrations.

ASM does not own the copyrights to Supplemental Material that may be linked to, or accessed through, an article. The authors have granted ASM a non-exclusive, world-wide license to publish the Supplemental Material files. Please contact the corresponding author directly for reuse.
