# Supplementary material for: Exogenous methionine contributes to reversing the resistance of Streptococcus suis to macrolides
Source: Microbiol Spectr. 2024 Jan 17;12(2):e02803-23. doi: 10.1128/spectrum.02803-23 (PMC10923279; doi:10.1128/spectrum.02803-23)
Supplement: Tables S1 to S4 — Primers used for the quantitative RT-PCR analysis and differential metabolites. [file spectrum.02803-23-s0001.docx]

**Table S1.** Primers used for the quantitative RT-PCR analysis.

| Genes | Primer sequence |
| --- | --- |
| *metK* | CGGGATACGATTGCGGAGATTGG |
|  | TGGCGATTGCTCTACAAGTGACG |
| *metE* | CCAGCCTACCAGTTGATGCCATC |
|  | TACCAGCGTAGAGGGTCTTGTCAG |
| *mtnN* | TGTTGCTGAAGGTATTGCCATCGG |
|  | CCCTGCCATTTGCCCGTAAGC |
| *luxS* | CCTCCCAAGTGATGCCTTCTGC |
|  | CTTTCGGGTGTCGGACAGGTTTC |
| *satA* | GCTCATTCGACCAACCGTAT |
|  | CACCACTTGGGTCAAGGACT |
| *satB* | TCGGCTCCGTATCGTGT |
|  | TCGGTGGCTTTACTTCC |
| *gyrA* | CCAGCCAGTGAGTATCCAACCAAAG |
|  | TGTCAAACCTGCCAGAGAACCATTC |
| *tetM* | GGGGATTCCCACAATCTTTT |
|  | ACATCCCATGCTCAGGTTC |
| *tetL* | TCTATATGGAGCTACCTGTCTGGATGG |
|  | AAGACTGACTATAGCCTGCACATTTCG |
| *tetW* | ATTCAAGCGGCAGTCACTTCCTTC |
|  | AAACAGCCAAAGAGCGGTACACC |
| *ermB* | CCGAACACTAGGGTTGCTCTTGC |
|  | AACATCTGTGGTATGGCGGGTAAG |
| *msrD* | ATGAGCGGTGGTGAAGAAACAAGG |
|  | GGTCTAAATGGCTCGTAGGTTCATCC |
| *msrAB* | TGGAGCCACTCGCACATTACATTC |
|  | GGTAGTCCGCAACATCAATCAGAGG |
| *dpr* | TGGTAGAGGTGTTCCGCTATCTGG |
|  | TGGTTACGCTGTCACCTTCTTCATC |
| *perR* | GGCACGAGCACCTCAATGTTATTTG |
|  | ACCAGTTTGGGTATGAGCTTCCTTC |
| *metQ* | GTAGACATCAACGCCTTCCAGCAC |
|  | TCCGCAATAGCAACCAAGTCTTCAC |
| *pnuC* | AACGGGTTGCCAGAGATTA |
|  | GGCGATAACAATAGGAAGGC |
| *msmK* | GAGCAAATCGGTAGCCCAGAAGAG |
|  | ACCATCTCCAACAAGAACGCCATC |
| *feoB* | ACCTCTACCGTGAGCAA |
|  | CACTATCTGGACCAACCC |
| *sodA* | CAGCAGAATTGGCAGCAGATATTGATG |
|  | AACCAAGAAAGCCCAACCTGAACC |
| *16S rRNA* | GTTGCGAACGGGTGAGTAA |
|  | TCTCAGGTCGGCTATGTATCG |

**Table S2.** Differential metabolites of S and T-I-8 strains

| Description | VIP | Fold change | p-value |
| --- | --- | --- | --- |
| N-Acetyl-D-Glucosamine 6-Phosphate | 1.022521209 | 2.322284361 | 1.85352E-08 |
| Histamine | 1.200468238 | 2.848505308 | 3.3507E-08 |
| Argininosuccinic acid | 1.155596142 | 3.194068866 | 2.70914E-07 |
| Adenosine 5'-diphosphate (ADP) | 1.533111761 | 4.945985051 | 7.02648E-07 |
| ADP-ribose | 1.823758613 | 8.800024266 | 9.79389E-07 |
| Nicotinamide adenine dinucleotide phosphate (NADP) | 1.104187202 | 1.806409101 | 1.19797E-06 |
| UDP-D-Galactose | 3.286825343 | 17.02156785 | 1.45984E-06 |
| Uridine 5'-diphosphate (UDP) | 1.169103175 | 8.88966039 | 1.60068E-06 |
| S-Methyl-5'-thioadenosine | 12.83985515 | 7.0704185 | 1.87497E-06 |
| .beta.-Homoproline | 2.815455242 | 3.2396867 | 2.10867E-06 |
| Adenosine 3',5'-diphosphate (PAP) | 1.300010577 | 6.242548632 | 2.92289E-06 |
| Adenosine monophosphate (AMP) | 5.978275481 | 8.398499084 | 2.96648E-06 |
| Uridine 5'-monophosphate (UMP) | 3.061425697 | 4.838959709 | 8.87815E-06 |
| L-Glutamate | 8.271862419 | 2.307229799 | 9.97339E-06 |
| Nicotinamide adenine dinucleotide (NAD) | 8.359180512 | 13.56560063 | 1.15402E-05 |
| Cytidine 5'-diphosphate (CDP) | 1.219464616 | 5.929851171 | 1.27704E-05 |
| L-Fucose-1-phosphate | 2.10325284 | 2.804722912 | 1.4268E-05 |
| L-Aspartate | 2.664760988 | 4.414015869 | 6.27306E-05 |
| Lys-Leu | 1.251470002 | 0.347353813 | 6.97961E-05 |
| 2'-Deoxyadenosine 5'-monophosphate (dAMP) | 1.107861332 | 1.621854407 | 7.56038E-05 |
| Allocystathionine | 4.613534864 | 4.013364804 | 7.79223E-05 |
| Choline | 9.738329331 | 1.710111939 | 9.62952E-05 |
| 1-Aminocyclopropanecarboxylic acid | 2.909326035 | 2.183314847 | 9.67617E-05 |
| Adenosine 5'-triphosphate (ATP) | 1.022315772 | 6.87581595 | 9.74107E-05 |
| D-Proline | 6.813656586 | 0.483227301 | 0.000101576 |
| Adenine | 2.487958942 | 3.750917299 | 0.000158181 |
| N-Acetylcadaverine | 13.47169351 | 26.59417534 | 0.000203907 |
| Glutathione disulfide | 1.889739168 | 4.980784423 | 0.000224953 |
| N-Acetylputrescine | 4.18466398 | 8.931097196 | 0.000232612 |
| Met-Ser | 1.571530915 | 3.66509641 | 0.00029468 |
| alpha-D-Glucose 1-phosphate | 1.221267081 | 2.999511233 | 0.000326122 |
| Reduced nicotinamide adenine dinucleotide (NADH) | 1.663471975 | 10.36082609 | 0.000342227 |
| D-Fructose 1,6-bisphosphate | 2.204635812 | 0.202145794 | 0.000358099 |
| Guanosine 5'-monophosphate (GMP) | 1.957974517 | 5.441789735 | 0.000368749 |
| EDTA | 1.198644541 | 2.44673056 | 0.000547219 |
| Isopentenyladenosine | 1.94063811 | 0.44675943 | 0.000587411 |
| Phosphorylcholine | 1.785552882 | 2.569383001 | 0.000612675 |
| Pyridoxine | 3.256086437 | 2.941488658 | 0.000688706 |
| Xanthylic acid (XMP) | 2.919833171 | 11.30522046 | 0.000988228 |
| Phthalic acid Mono-2-ethylhexyl Ester | 3.452153886 | 1.441656744 | 0.00099165 |
| Norepinephrine | 2.294798965 | 3.042293236 | 0.001750481 |
| Oxypurinol | 2.088177398 | 4.547857854 | 0.001815238 |
| L-Carnitine | 6.310654116 | 1.670737524 | 0.002384638 |
| 2-Hydroxyadenine | 4.941924437 | 0.745486215 | 0.002455905 |
| D-glucosamine 6-phosphate | 1.951360358 | 1.656339111 | 0.002671856 |
| N-Acetyl-L-glutamate | 1.266958836 | 10.26829224 | 0.002921576 |
| N-Acetyl-L-Histidine | 1.71758565 | 3.896815482 | 0.003117299 |
| 1,2-Benzenedicarboxylic acid | 2.497453698 | 10.8883558 | 0.003422801 |
| Vanillin | 1.36793182 | 0.224989459 | 0.003753615 |
| Dioctyl phthalate | 8.705602379 | 27.50953263 | 0.0039042 |
| Dimethylaminopurine | 1.357567275 | 0.497391487 | 0.003937963 |
| L-Asparagine | 1.097325592 | 1.667259433 | 0.00431127 |
| 2'-Deoxyguanosine 5'-diphosphate (dGDP) | 1.030886805 | 3.222496883 | 0.004535888 |
| L-Histidine | 4.577046683 | 0.606821618 | 0.005642982 |
| Thioetheramide-PC | 1.894190443 | 3.105273472 | 0.005664414 |
| Xanthosine | 1.391288553 | 3.249881799 | 0.005786228 |
| Ile-Val | 1.501985664 | 5.299360968 | 0.006186119 |
| Cytidine 5'-monophosphate | 3.037453148 | 4.547900028 | 0.006376173 |
| N-Acetylneuraminic acid | 2.843565706 | 1.85683679 | 0.007235136 |
| Uridine | 1.975336454 | 1.73808631 | 0.007246999 |
| Cytidine | 2.863420603 | 4.709635392 | 0.008807889 |
| L-Citrulline | 5.448881319 | 3.536004186 | 0.009100267 |
| Ornithine | 1.060043842 | 1.286399641 | 0.009975154 |
| Glycerophosphocholine | 6.405963545 | 1.886122725 | 0.010474633 |
| N6-Methyladenine | 1.534835881 | 0.34841366 | 0.011780289 |
| 5-Methylcytidine | 1.391544534 | 0.265958075 | 0.013142501 |
| 1-Methyladenosine | 1.428499868 | 1.797755196 | 0.013668825 |
| D-Pipecolinic acid | 1.014871553 | 0.669178038 | 0.013971318 |
| Adenosine | 14.45543016 | 4.416343881 | 0.01401605 |
| Cytosine | 2.405886213 | 2.545162418 | 0.014391625 |
| Nicotinamide | 4.273157832 | 1.712980144 | 0.015919966 |
| Tylosin | 4.102995274 | 9.26036569 | 0.019252635 |
| N6,N6,N6-Trimethyl-L-lysine | 1.65020595 | 0.550290118 | 0.019550279 |
| S-Adenosylmethionine | 1.251576855 | 1.726308082 | 0.021848768 |
| (3-Carboxypropyl)trimethylammonium cation | 1.112604185 | 1.521259523 | 0.023271953 |
| His-Pro | 5.181725727 | 1.958128885 | 0.024690566 |
| His-Ala | 1.230443949 | 1.943962574 | 0.028054799 |
| Hypoxanthine | 15.88229395 | 0.696680866 | 0.034529006 |
| Genistein | 2.067871412 | 0.524853718 | 0.041540193 |
| L-Pyroglutamic acid | 1.430014613 | 0.707857621 | 0.043039702 |
| N2-Acetyl-L-ornithine | 4.756472932 | 0.602339824 | 0.04871701 |

**Table S3.** Differential metabolites of S and T-I-64 strains.

| Description | VIP | Fold change | p-value |
| --- | --- | --- | --- |
| N-Acetyl-L-glutamate | 1.171491349 | 10.26658501 | 1.0126E-10 |
| N-Acetylglutamine | 1.857668862 | 11.97008298 | 3.59069E-10 |
| N-Acetylcadaverine | 4.243946592 | 3.745466133 | 6.54606E-10 |
| Lys-Asp | 1.583153898 | 3.867037404 | 6.51104E-09 |
| Glutathione disulfide | 2.598755278 | 8.607300356 | 6.76607E-09 |
| 2'-Deoxyadenosine 5'-monophosphate (dAMP) | 4.002051553 | 6.76333974 | 1.65654E-08 |
| N-Acetyl-L-Histidine | 2.354990931 | 6.214517782 | 2.41475E-08 |
| Adenosine monophosphate (AMP) | 4.451150217 | 5.68691409 | 2.863E-08 |
| Nicotinamide adenine dinucleotide phosphate (NADP) | 1.177841115 | 1.831469322 | 3.18475E-08 |
| Guanosine 5'-monophosphate (GMP) | 1.802287092 | 5.051659121 | 4.1069E-08 |
| L-Fucose-1-phosphate | 2.041539975 | 2.832988059 | 5.02732E-08 |
| Acetylcarnitine | 1.195185512 | 7.74492861 | 5.30803E-08 |
| Flavin adenine dinucleotide (FAD) | 2.16432344 | 3.857332934 | 8.01045E-08 |
| 6-Phospho-D-gluconate | 1.084800292 | 3.70813823 | 9.39545E-08 |
| Uridine 5'-monophosphate (UMP) | 2.468024094 | 3.741495146 | 1.50183E-07 |
| Xanthylic acid (XMP) | 1.593488818 | 4.151605028 | 1.71698E-07 |
| Glu-Pro | 1.447015724 | 2.382565359 | 2.02624E-07 |
| Uridine 5'-diphosphate (UDP) | 1.153908801 | 4.315573613 | 2.04625E-07 |
| L-Aspartate | 2.162442 | 3.278483613 | 2.5112E-07 |
| 2'-Deoxyuridine | 2.21518751 | 4.693681054 | 3.25558E-07 |
| S-Methyl-5'-thioadenosine | 12.36409768 | 7.126349402 | 3.2982E-07 |
| 1,2-Benzenedicarboxylic acid | 2.040655215 | 7.93184306 | 3.44677E-07 |
| Adenosine 5'-diphosphate (ADP) | 1.395893888 | 4.636177633 | 4.81028E-07 |
| Pro-Thr | 2.458623638 | 3.161129795 | 5.75494E-07 |
| Cytidine 5'-diphosphate (CDP) | 1.076122559 | 5.346445858 | 8.79192E-07 |
| 2-Butoxyethanol | 1.295871938 | 3.466177623 | 9.38854E-07 |
| Oxypurinol | 3.444439571 | 10.56428712 | 1.18458E-06 |
| Cytidine 5'-monophosphate | 3.254367537 | 4.859434706 | 1.44749E-06 |
| Deoxythymidine 5'-phosphate (dTMP) | 2.387960302 | 4.185333242 | 1.65905E-06 |
| Xanthosine | 2.831907521 | 9.66399789 | 1.70962E-06 |
| Glycerol 3-phosphate | 1.02860026 | 4.570432257 | 1.99187E-06 |
| Dioctyl phthalate | 7.024054566 | 19.30050925 | 2.5068E-06 |
| Thymidine 5'-monophosphate | 1.454383761 | 3.93751142 | 3.0381E-06 |
| gamma-L-Glutamyl-L-glutamic acid | 1.3806976 | 2.148247235 | 3.21143E-06 |
| 3-Methylhistidine | 1.264844366 | 3.86385152 | 3.60175E-06 |
| Ile-Val | 1.283996614 | 3.638910814 | 3.99204E-06 |
| L-Pyroglutamic acid | 1.110586093 | 3.19649544 | 4.00148E-06 |
| Pro-Asn | 1.445186861 | 2.55579665 | 4.33298E-06 |
| Uridine | 3.790130718 | 2.667893684 | 4.45127E-06 |
| Betaine | 7.090068362 | 1.815930753 | 8.84257E-06 |
| Deoxyadenosine | 5.67643821 | 4.878376929 | 1.30849E-05 |
| Val-Glu | 1.047938745 | 2.238683492 | 2.05077E-05 |
| Adenosine | 14.9274261 | 4.363550842 | 2.56019E-05 |
| EDTA | 1.45314225 | 2.810902923 | 2.85083E-05 |
| .beta.-Homoproline | 2.560801701 | 3.022697749 | 3.40716E-05 |
| Lys-Leu | 1.100223269 | 0.336662127 | 4.49574E-05 |
| Adenine | 2.727143833 | 4.515406963 | 4.78884E-05 |
| 1-methylguanosine | 1.147376864 | 2.355653162 | 5.85758E-05 |
| N-Acetyl-D-glucosamine | 2.913449244 | 9.038926235 | 7.40632E-05 |
| 4-Imidazoleacetic acid | 1.519057736 | 0.596026239 | 9.78561E-05 |
| Glycerophosphocholine | 7.098209157 | 1.918921557 | 0.000103889 |
| Ergothioneine | 8.657836442 | 0.175110372 | 0.000105482 |
| Ser-Asp | 1.261725378 | 4.991753819 | 0.000110854 |
| PC(16:0/16:0) | 1.77125767 | 61.67004072 | 0.000131276 |
| Guanosine | 6.428578306 | 1.577342975 | 0.000137473 |
| Cytidine | 2.688566229 | 4.182435101 | 0.000169492 |
| Choline | 7.449656156 | 1.408587716 | 0.000180491 |
| Uracil | 4.13076179 | 1.77532016 | 0.000234673 |
| Nicotinamide adenine dinucleotide (NAD) | 2.353880979 | 2.260066539 | 0.000238071 |
| N-Acetylputrescine | 1.257890462 | 1.896131502 | 0.000241858 |
| beta-Nicotinamide D-ribonucleotide | 5.439757377 | 0.536902256 | 0.000326341 |
| L-Citrulline | 2.366020157 | 0.525284459 | 0.000573708 |
| Pro-Glu | 1.068658404 | 1.59162137 | 0.000588623 |
| Genistein | 2.325022986 | 0.20801868 | 0.000607964 |
| L-Glutamate | 3.866524718 | 1.334919839 | 0.000696326 |
| S-Adenosylmethionine | 1.631375936 | 1.930817781 | 0.000719267 |
| Cytosine | 2.246933411 | 2.279786511 | 0.000723859 |
| Phosphorylcholine | 1.248062439 | 1.799104035 | 0.001198726 |
| 1-Aminocyclopropanecarboxylic acid | 1.35500853 | 1.289755717 | 0.001298497 |
| Nicotinamide ribotide | 2.275622847 | 0.693455846 | 0.001626972 |
| 1-Methyladenosine | 4.452822695 | 7.232303478 | 0.001949347 |
| D-Fructose 1,6-bisphosphate | 1.491376022 | 0.384213262 | 0.002216922 |
| L-Carnitine | 4.523274438 | 1.310994631 | 0.002270513 |
| Nicotinamide | 4.469384007 | 0.45358053 | 0.003025587 |
| Agomelatine | 1.22632861 | 1.297392811 | 0.003618037 |
| 5-Methylcytidine | 1.269823355 | 0.103425899 | 0.003891371 |
| L-Methionine | 1.591749366 | 0.473527031 | 0.004299033 |
| D-Pipecolinic acid | 2.899697976 | 0.703384594 | 0.004805147 |
| Thymine | 1.133565166 | 1.801146313 | 0.005001762 |
| D-Proline | 3.158885019 | 0.597472006 | 0.005471587 |
| NG,NG-dimethyl-L-arginine(ADMA) | 1.337270737 | 5.016890963 | 0.005662092 |
| DL-Indole-3-lactic acid | 3.347671169 | 0.490536495 | 0.005664498 |
| L-Tryptophan | 2.305947864 | 0.465079715 | 0.005821679 |
| Harmane | 2.799976752 | 2.164289619 | 0.007487132 |
| Triethanolamine | 2.021763192 | 1.684742025 | 0.008268177 |
| 2-Hydroxyadenine | 3.685161649 | 1.28741173 | 0.0087847 |
| Allopurinol riboside | 3.456544692 | 1.2370331 | 0.008852049 |
| Allocystathionine | 1.345389784 | 1.859477956 | 0.009663338 |
| Phthalic acid Mono-2-ethylhexyl Ester | 3.181512563 | 1.31075509 | 0.00977181 |
| 1-Stearoyl-2-oleoyl-sn-glycerol 3-phosphocholine (SOPC) | 2.202025763 | 80.42455208 | 0.01028871 |
| L-Histidine | 3.384180222 | 0.668664155 | 0.013902042 |
| His-Met | 1.074220832 | 4.453689922 | 0.014043333 |
| Isopentenyladenosine | 1.302274392 | 1.412753222 | 0.014261377 |
| 3-Phospho-D-glycerate | 1.042710943 | 2.350235744 | 0.014499195 |
| Dimethylaminopurine | 1.048861257 | 0.560554444 | 0.014845554 |
| Myristoleic acid | 1.983381314 | 11.37193175 | 0.016220363 |
| D-glucosamine 6-phosphate | 1.237338847 | 0.716856683 | 0.026307074 |
| Ornithine | 1.841216206 | 0.68857398 | 0.029039809 |
| Tyramine | 5.640822423 | 0.584857748 | 0.030812664 |
| Norharmane | 1.750200795 | 1.721505932 | 0.031637086 |
| Trigonelline | 1.12809489 | 0.594603833 | 0.040341882 |
| Pro-Ser | 1.190514141 | 1.935062497 | 0.04147567 |
| L-Phenylalanine | 4.327792698 | 0.617984084 | 0.043059646 |
| L-Leucine | 1.538584781 | 1.759474078 | 0.045539613 |
| Hypoxanthine | 2.27909368 | 1.485611656 | 0.048795059 |

**Table S4.** Differential metabolites of S and T-I-256 strains

| description | VIP | Fold change | p-value |
| --- | --- | --- | --- |
| L-Citrulline | 3.253028751 | 0.223443162 | 9.18386E-08 |
| Isopentenyladenosine | 2.090353137 | 0.214308371 | 1.00383E-06 |
| Allopurinol riboside | 6.093275766 | 0.494612997 | 2.92757E-06 |
| N-Acetyl-L-Histidine | 1.214710794 | 2.658453025 | 4.41249E-06 |
| D-Glucose 6-phosphate | 1.032660667 | 0.437375729 | 6.24826E-06 |
| Betaine | 3.282517766 | 0.754593163 | 2.75857E-05 |
| Guanosine | 6.102366347 | 0.554708361 | 3.49761E-05 |
| D-Proline | 6.110517442 | 0.460416824 | 6.42018E-05 |
| Dimethylaminopurine | 1.473704137 | 0.291846903 | 0.000194277 |
| D-Fructose 1,6-bisphosphate | 1.99906427 | 0.152432592 | 0.000237431 |
| N-Acetylneuraminic acid | 1.549116265 | 0.532114731 | 0.000251264 |
| L-Tyrosine | 2.99412801 | 0.388175203 | 0.000294504 |
| Cytidine monophosphate N-acetylneuraminic acid | 7.343284331 | 0.485995208 | 0.000312115 |
| L-Methionine | 1.789024472 | 0.478270847 | 0.000384802 |
| trans-2-Hydroxycinnamic acid | 2.476695446 | 0.409311379 | 0.000515212 |
| N-Acetylcadaverine | 4.908474425 | 6.188614999 | 0.000539637 |
| Cytidine 5'-monophosphate | 5.266725094 | 0.514366536 | 0.000556791 |
| L-Threonine | 1.334452511 | 0.44645742 | 0.000567775 |
| Dopamine | 2.14651903 | 0.404261498 | 0.000576463 |
| beta-Nicotinamide D-ribonucleotide | 5.572521465 | 0.528069349 | 0.000680556 |
| Ornithine | 2.284933608 | 0.606223276 | 0.001054638 |
| Atrolactic acid | 1.015895659 | 0.369499894 | 0.001082269 |
| DL-O-tyrosine | 1.006751061 | 0.494955205 | 0.001289419 |
| Vanillin | 1.293875734 | 0.11666414 | 0.001465139 |
| L-Phenylalanine | 6.110837209 | 0.333308 | 0.001466609 |
| Tyramine | 7.670873822 | 0.32147755 | 0.001526373 |
| Nicotinamide adenine dinucleotide (NAD) | 3.225635198 | 3.841712779 | 0.001561502 |
| DL-Indole-3-lactic acid | 3.631639787 | 0.452683744 | 0.001645802 |
| S-Methyl-5'-thioadenosine | 7.34580017 | 3.731505443 | 0.001771828 |
| L-Tryptophan | 2.461815424 | 0.425602171 | 0.001935138 |
| Pro-Thr | 1.424205379 | 2.016922136 | 0.001966234 |
| Cytosine | 1.250518355 | 0.648165861 | 0.002025186 |
| N-Acetylputrescine | 1.150977775 | 2.145379675 | 0.002499672 |
| D-Pipecolinic acid | 3.18653593 | 0.7287515 | 0.003282816 |
| Dioctyl phthalate | 5.943392263 | 16.99535351 | 0.003601776 |
| 4-Imidazoleacetic acid | 1.762573822 | 0.429093147 | 0.003910405 |
| Ergothioneine | 11.28839224 | 2.765123022 | 0.00532535 |
| S-Adenosylmethionine | 1.01408652 | 1.636499899 | 0.005883194 |
| Thymine | 1.19886567 | 1.52116709 | 0.005940029 |
| 1-Methyladenosine | 1.479324876 | 0.338578342 | 0.006044674 |
| D-Alanyl-D-alanine (D-Ala-D-Ala) | 3.263818458 | 0.422371655 | 0.00659784 |
| Adenosine | 15.62852474 | 5.713700139 | 0.006712219 |
| Genistein | 1.940639421 | 0.403355759 | 0.007846397 |
| N6-Methyladenine | 1.342230042 | 0.30136797 | 0.00790669 |
| D-glucosamine 6-phosphate | 1.5393829 | 0.609307836 | 0.007974504 |
| 1,2-Benzenedicarboxylic acid | 1.630179984 | 6.758019164 | 0.008219912 |
| Hypoxanthine | 16.62989354 | 0.667299212 | 0.00834158 |
| 2-Hydroxyadenine | 7.985725646 | 0.571412274 | 0.00862578 |
| DL-Methionine sulfoxide | 1.885213295 | 0.24209008 | 0.008682309 |
| Pro-Asn | 2.705564678 | 0.52274926 | 0.008900885 |
| Uridine | 1.162878477 | 0.560049515 | 0.009450313 |
| 6-Aminocaproic acid | 1.250342677 | 1.835059536 | 0.010971852 |
| Allocystathionine | 1.346797422 | 1.969242041 | 0.011198401 |
| Adenosine monophosphate (AMP) | 1.556010321 | 1.980947197 | 0.011387376 |
| D-Mannose 1-phosphate | 1.183599855 | 0.526767579 | 0.011495772 |
| Deoxyadenosine | 3.084756785 | 2.621851235 | 0.012107253 |
| Adenine | 1.427003044 | 2.239811405 | 0.014524201 |
| Nicotinamide | 1.635670611 | 0.683472168 | 0.014929666 |
| Glu-Ser | 1.505462095 | 0.602096911 | 0.01790759 |
| Uracil | 2.540322081 | 0.753661987 | 0.019114472 |
| Phthalic acid Mono-2-ethylhexyl Ester | 2.10581471 | 1.271879255 | 0.020946779 |
| L-Carnitine | 4.444653289 | 0.727802731 | 0.02211731 |
| Thioetheramide-PC | 1.790483259 | 3.337030848 | 0.02300853 |
| EDTA | 1.365639274 | 3.279754463 | 0.023341348 |
| 3-Aminobutanoic acid | 2.265459019 | 0.482136077 | 0.024178913 |
| Glu-His | 1.093052894 | 3.278218411 | 0.026161658 |
| His-Ser | 1.299970034 | 0.74171617 | 0.029733348 |
| 1-Stearoyl-2-oleoyl-sn-glycerol 3-phosphocholine (SOPC) | 1.165900873 | 22.69055203 | 0.035702996 |
| N6,N6,N6-Trimethyl-L-lysine | 1.33185899 | 0.621710087 | 0.037219177 |
| L-Leucine | 1.676212012 | 2.212754722 | 0.043652156 |
| 5-Methylcytidine | 1.033050353 | 0.425000061 | 0.043738723 |
| Nicotinamide ribotide | 2.033314368 | 0.775803332 | 0.044025893 |
| Trigonelline | 1.031477651 | 0.611768971 | 0.04410382 |
