# Supplementary material for: Exogenous methionine contributes to reversing the resistance of Streptococcus suis to macrolides
Source: Microbiol Spectr. 2024 Jan 17;12(2):e02803-23. doi: 10.1128/spectrum.02803-23 (PMC10923279; doi:10.1128/spectrum.02803-23)
Supplement: Fig. S1 to S4 — Fig S1 to S3 (Bioinformatics analysis of the metabolomics of S. suis) and Fig S4 (The time-kill curves of Streptococcus suis treated with tylosin and methionine at different concentrations. [file spectrum.02803-23-s0002.pdf]

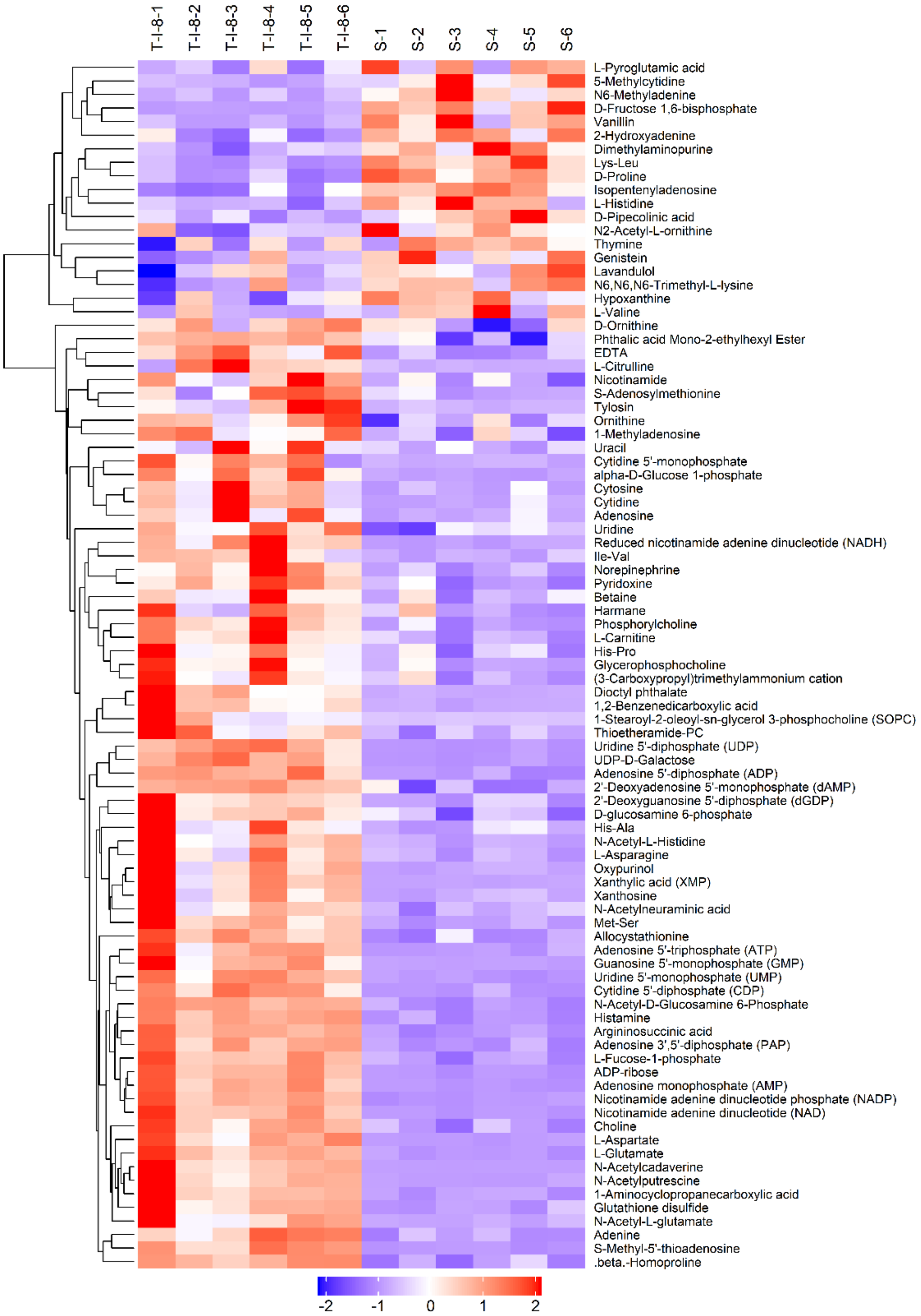

Figure S1 Overview metabolic profile heat map shows the comparison of control group S with *S. suis* T-I-8 in this study.

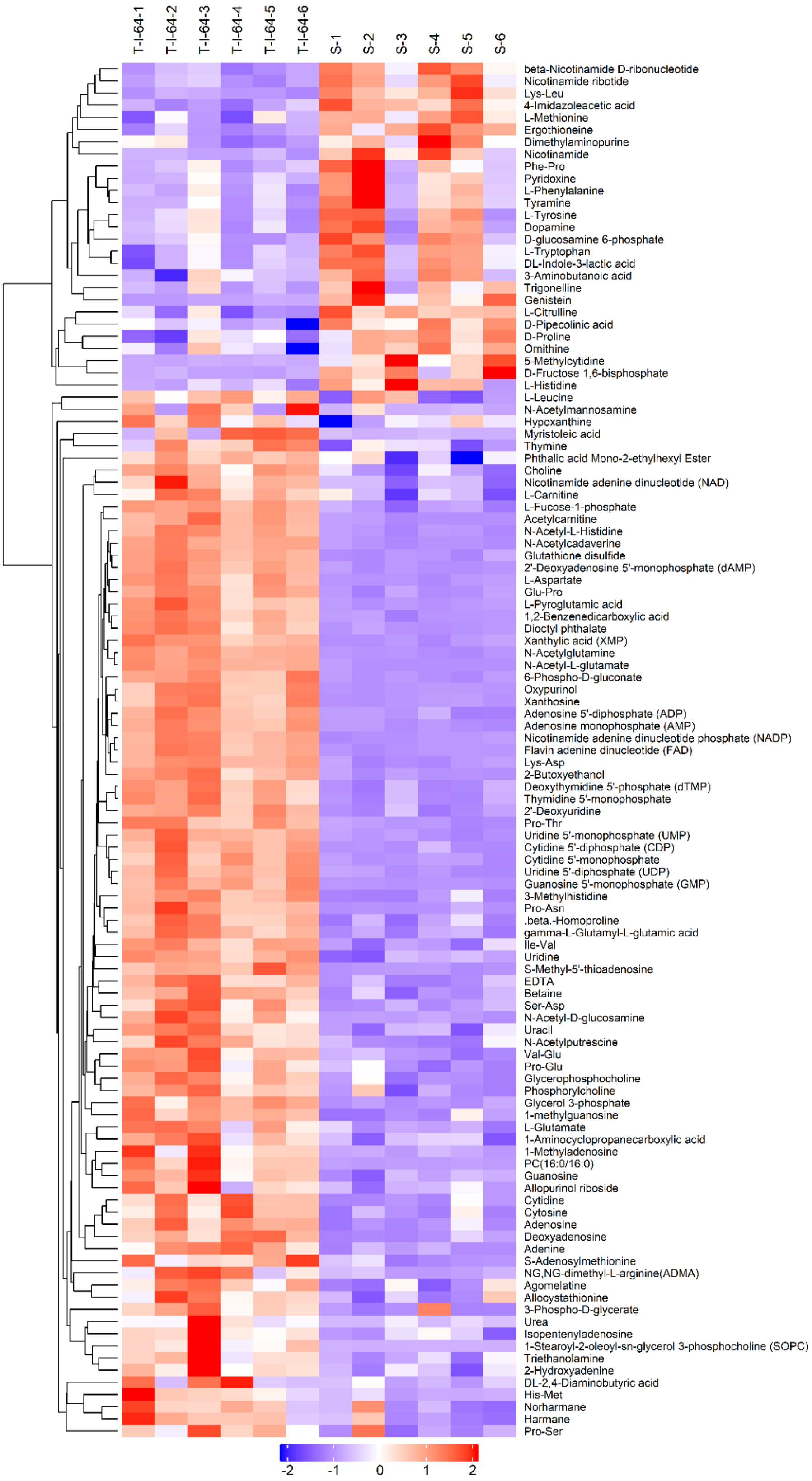

Figure S2 Overview metabolic profile heat map shows the comparison of control group S with *S. suis* T-I-64 in this study.

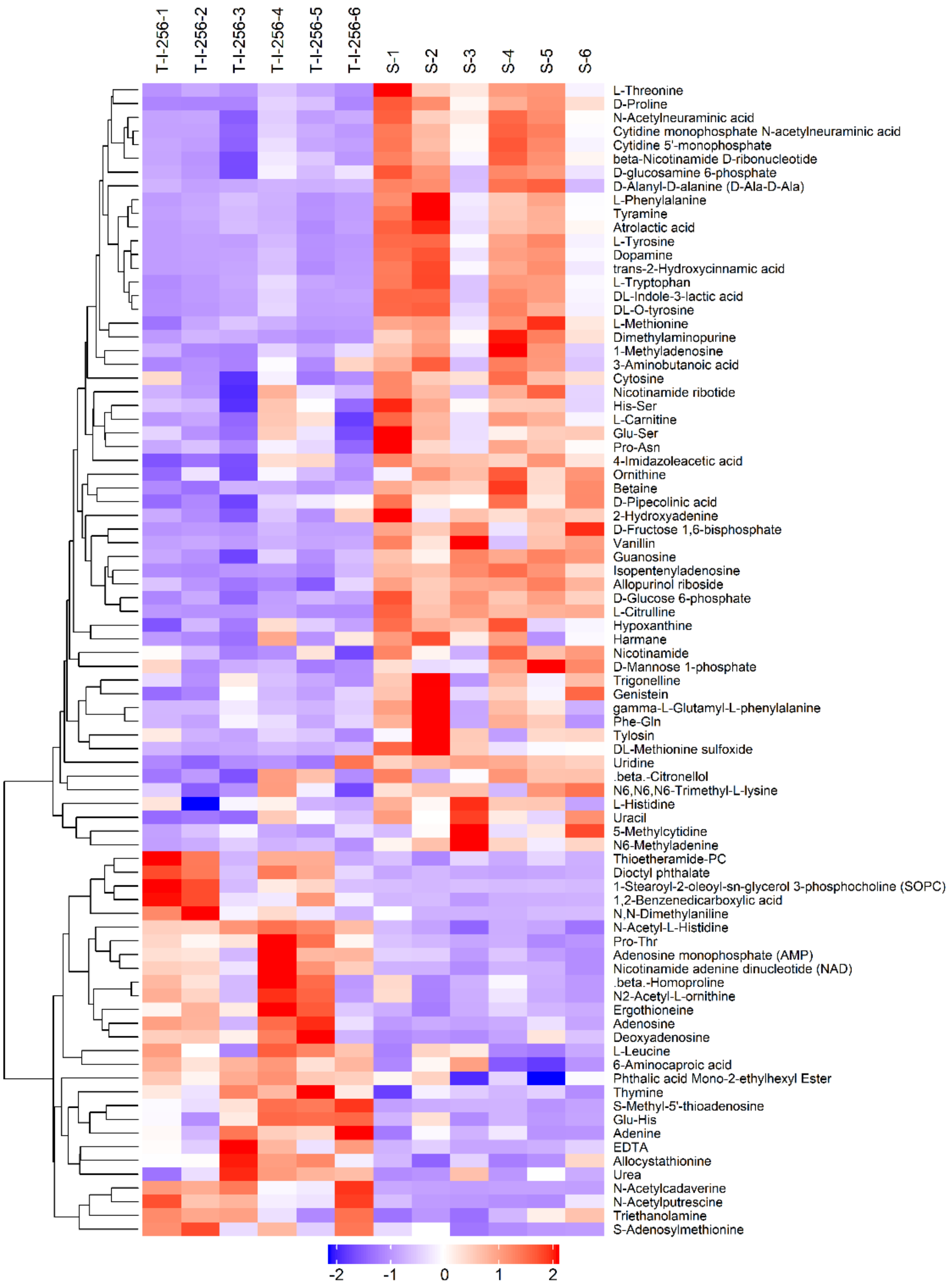

Figure S3 Overview metabolic profile heat map shows the comparison of control group S with *S. suis* T-I-256 in this study.

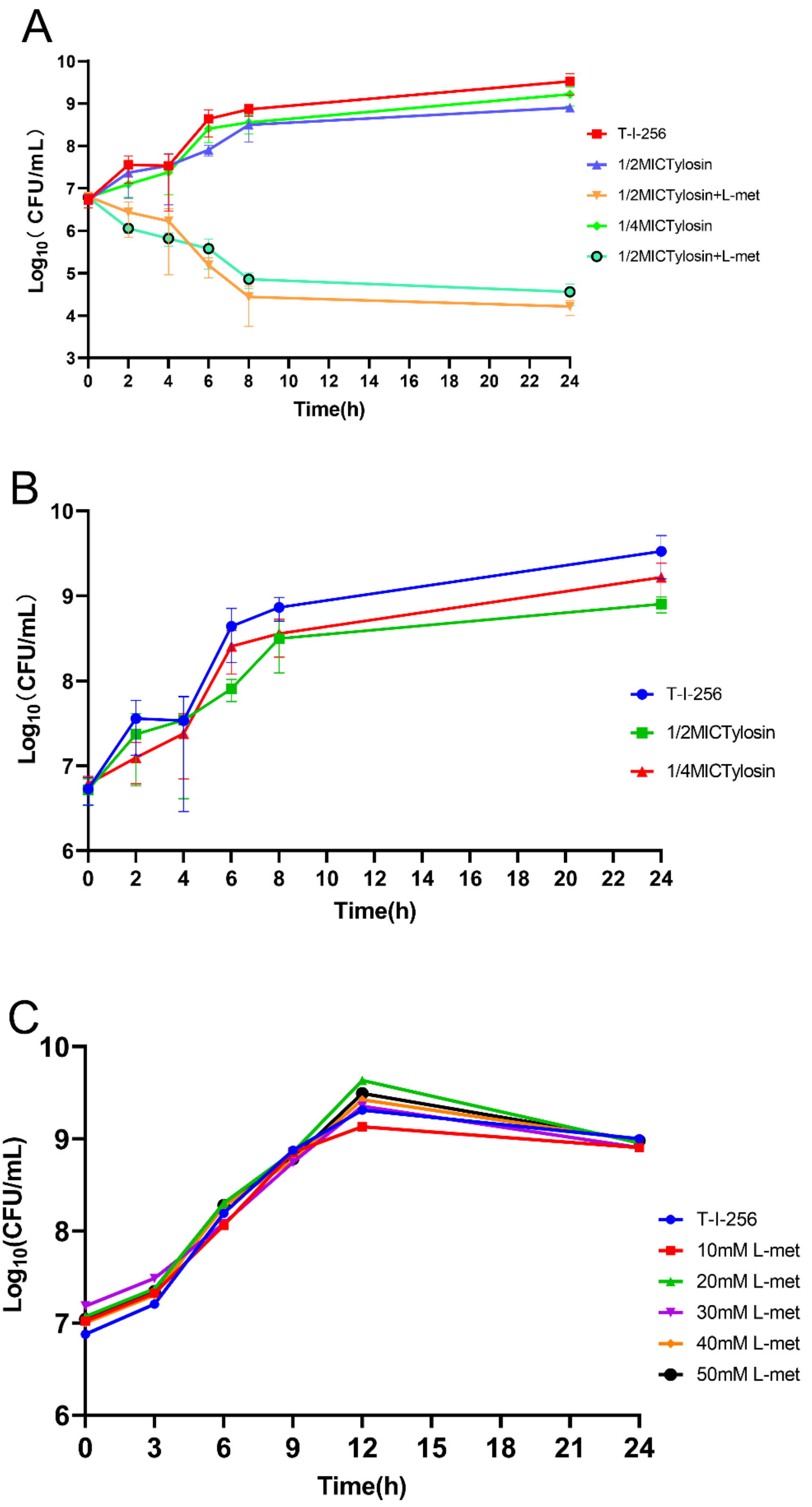

Figure S4 The time-kill curves of *Streptococcus suis* T-I-256. (A) The effect of 50mM methionine combination with 1/2 MIC or 1/4 MIC tylosin on *S. suis* T-I-256. (B) The effect of 1/2 MIC or 1/4 MIC tylosin alone on *S. suis* T-I-256. (C) The effect of different concentrations of methionine alone on *S. suis* T-I-256.
